# Sedentism and plant cultivation in northeast China emerged during affluent conditions

**DOI:** 10.1371/journal.pone.0218751

**Published:** 2019-07-18

**Authors:** Gideon Shelach-Lavi, Mingyu Teng, Yonaton Goldsmith, Ido Wachtel, Chris J. Stevens, Ofer Marder, Xiongfei Wan, Xiaohong Wu, Dongdong Tu, Roi Shavit, Pratigya Polissar, Hai Xu, Dorian Q. Fuller

**Affiliations:** 1 Department of Asian Studies, The Hebrew University of Jerusalem, Jerusalem, Israel; 2 Center for Frontier Archaeology, Jilin University, Changchun, China; 3 Department of Geological and Planetary Sciences, California Institute of Technology, Pasadena, CA, United States of America; 4 Institute of Earth Sciences, The Hebrew University of Jerusalem, Jerusalem, Israel; 5 Department of Archaeology, The Hebrew University of Jerusalem, Jerusalem, Israel; 6 Institute of Archaeology, University College London, London, England; 7 Department of Bible Studies, Archeology and the Ancient Near East, Ben-Gurion University, Beer Sheva, Israel; 8 Institute of Archaeology and Cultural Relics, Liaoning Province, Shanyang, China; 9 School of Archaeology and Museology, Peking University, Beijing, China; 10 Lamont Doherty Earth Observatory, Palisades, NY, United States of America; 11 Institute of Surface-Earth System Science, Tianjin University, Tianjin, China; Institute of Geographic Sciences and Natural Resources Research Chinese Academy of Sciences, CHINA

## Abstract

The reasons and processes that led hunter-gatherers to transition into a sedentary and agricultural way of life are a fundamental unresolved question of human history. Here we present results of excavations of two single-occupation early Neolithic sites (dated to 7.9 and 7.4 ka) and two high-resolution archaeological surveys in northeast China, which capture the earliest stages of sedentism and millet cultivation in the second oldest center of domestication in the Old World. The transition to sedentism coincided with a significant transition to wetter conditions in north China, at 8.1–7.9 ka. We suggest that these wetter conditions were an empirical precondition that facilitated the complex transitional process to sedentism and eventually millet domestication in north China. Interestingly, sedentism and plant domestication followed different trajectories. The sedentary way of life and cultural norms evolved rapidly, within a few hundred years, we find complex sedentary villages inhabiting the landscape. However, the process of plant domestication, progressed slowly over several millennia. Our earliest evidence for the beginning of the domestication process appear in the context of an already complex sedentary village (late Xinglongwa culture), a half millennia after the onset of cultivation, and even in this phase domesticated plants and animals were rare, suggesting that the transition to domesticated (*sensu stricto*) plants in affluent areas might have not played a substantial role in the transition to sedentary societies.

## Introduction

The transition to a sedentary way of life and the domestication of plants is arguably the most significant ‘revolution’ in human history. This transition gradually resulted in a dramatic increase of population size and density, in craft specialization and the division of labor and the initiation of social dynamics and the accumulation of resources that are linked to the development of socio-political stratification [1,2].

The processes, mechanisms and possible drivers of a transition from one subsistence mode to another have long been at the forefront of anthropological and archaeological research. Three hypotheses have been proposed to explain such transitions. The first suggests that stress, induced either by deteriorating environmental conditions (e.g., the ‘oasis hypothesis’ [3, 4]) or by population increase led to an overexploitation of the environment and forced innovation [5]. A current example is the optimal foraging theory [6] and the diet breadth model [7], which predict that a transition to a delayed returns system (i.e. long-term tending resources) will occur during times of scarce resources, promoting a border dietary range. The second hypothesis argues that transitions occur at times of enhanced resources [8,9]. A current example is the cultural niche construction theory [10], which claims that organisms shape their environment during stable or affluent times, these allow experimentation with locally available resources, which improve crop yield and eventually lead to domestication [11]. The third hypothesis argues that transitions occur as a result of socioeconomic competition and are not affected by external forcings [12,13].

Here, we present evidence for the timing, duration and environmental conditions of the onset of sedentism and plant cultivation in northern China the second oldest center of domestication in the Old World. We address the first two hypotheses by examining whether the onset of sedentism and cultivation occurred at a time of stress or affluent conditions and whether these environmental conditions were stable or fluctuating. We assume that the limiting environmental parameter is rainfall and thus we focus on the timing of rainfall changes and human settlement patterns at the onset of sedentism and cultivation in north China. We acknowledge the possibility that temperature might have also played a role in restricting millet growth [14], however, as the magnitude, timing and seasonal structure of temperature change in north China in uncertain [14], we focus here on rainfall. The combination of high-resolution archaeological and paleoclimatic data enable to test the theoretical frameworks of the onset of sedentism, domestication and population increase.

In addition to addressing the processes leading to the onset of sedentism and plant cultivation, the combination of excavation and survey results enable us to follow the evolution and trajectories of these two innovations. We show that sedentism evolved rapidly, whereas, plant domestication progressed at a much slower pace.

## Millet domestication in northeast China

North China is one of a few centers in the world where complex agricultural systems emerged independently. The native staple grains are two types of millet (foxtail and broomcorn), however, the date, process and the paleoclimatic context of their domestication are debated [15–20]. We focus on northeast China because currently the earliest undisputed examples of domesticated millet grains and the most extensive evidence for early millet consumption have been found in this region, at sites of the Xinglongwa culture [21–23].

Early research in north China began in the 1970s and focused on the middle reaches of the Yellow River. It identified remains of early Neolithic sedentary societies of the 7^th^ and 6^th^ millennia BCE, which were sorted into a series of ‘cultures’, most notably Cishan and Peiligang. These findings focused attention to this area ‒ which classical Chinese tradition identifies as ‘the cradle of Chinese civilization’, the birthplace of agriculture from whence it spread to other regions in north and central China [24]. By the late 1980s, it became clear that contemporaneous sedentary societies also existed in other regions of north China, including the Houli culture in the lower Yellow River area and the Xinglongwa culture in Northeast China [25,26]. In recent years even earlier phases of the "Neolithic" period were identified in the different sub-regions of North China, such as the Xiaohexi phase in the northeast (24), but those are not well studied and are poorly dated. While substantial archaeological knowledge of sedentary prehistoric societies has been presented, the earlier phases that preceded the sedentary cultures and the transition process from mobile to sedentary societies remains mostly obscure [19]. To address these developments and their context during the important phase of the transition to agriculture, we integrate in this paper novel data from the excavations of two early Neolithic sites, the results of two high-resolution archaeological surveys in northeast China and novel paleo-climatic data.

## Results

### Systematic archaeological surveys in northeast China

**Fig 1:**
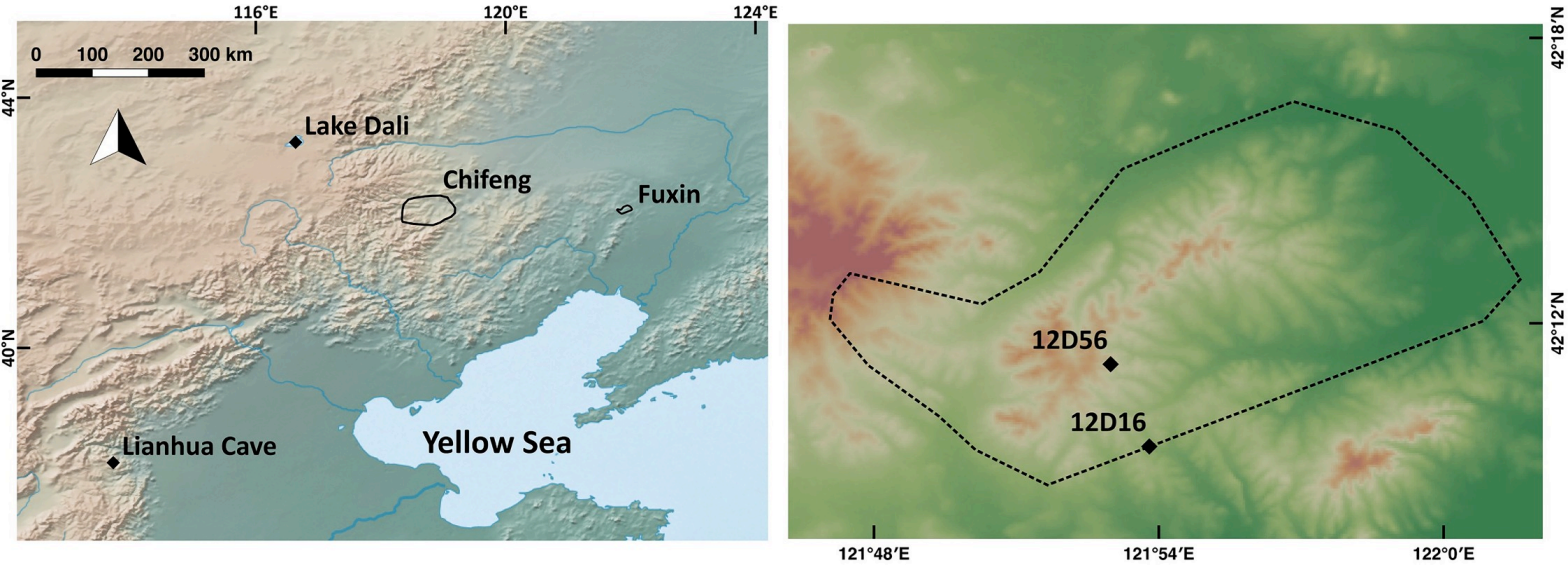
Location maps. Left: Map of the survey areas and the paleoclimate records discussed in the paper. Right: Topographic map of the Fuxin survey region, showing the borders of the survey and the two sites excavated.

**Fig 2:**
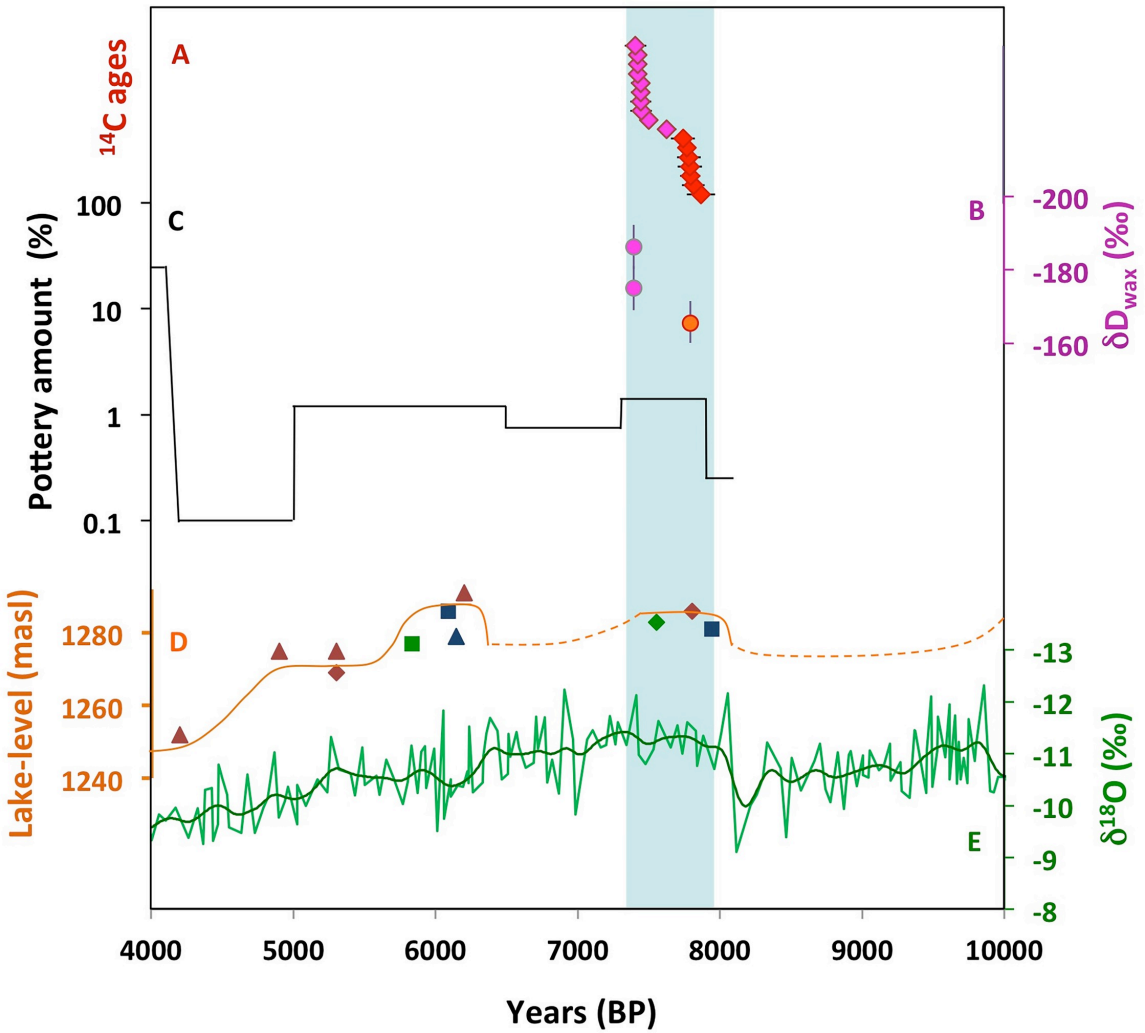
Paleoclimate of northeast China. a. Radiocarbon ages from both excavated sites (red–site 12D56, purple–site 12D16). b. ^avg^δD_wax_ from both sites (color same as 4a). c. Normalized pottery density from the two survey regions [28]. d. Lake Dali lake level (for symbols see [32]). e. Lianhua Cave δ^18^O (light green- redrawn from ref [33], dark green is a Gaussian smoothing of data). The vertical bar denotes the time span of both periods.

Over the past 20 years, four high-resolution, detailed, large-scale regional archaeological surveys were conducted in northeast China [27–30]. Here we focus on the surveys conducted around the modern cities of Chifeng and Fuxin [27,28](Fig 1) because these are the only surveys that recovered evidence of the earlier phases of sedentism. These surveys were designed to systematically recover data that enable population estimates and the reconstruction of local socio-political trajectories [31]. The full area was systematically surveyed on foot (at a 50 and 20 m resolution), the locations and densities of pottery shards and stone tools were mapped, counted and statistically analyzed. A total pottery amount was calculated for each period by integrating the area of the occupied territory, the density of artifacts and the length of each period. These results provide a direct reconstruction of population trajectory in these two regions and present a qualitative demographic history of northeast China (Fig 2C; S1 Text, S1 Fig). The earliest sites found in both regions are those attributed to the Xiaohexi phase and the Xinglongwa culture, which represents the earliest phase of sedentism in this region. The combined results of the two surveys demonstrate a rapid increase in population and the complexity of village society following the initial stage of sedentism (Fig 2C).

### Excavations of two Early Neolithic sites

To evaluate the timing and socio-economic processes involved in the transition to sedentism, we excavated two single-occupation sites discovered during our survey in the Fuxin area. Those sites capture the onset and earliest phases of sedentism (Fig 1).

**Fig 3:**
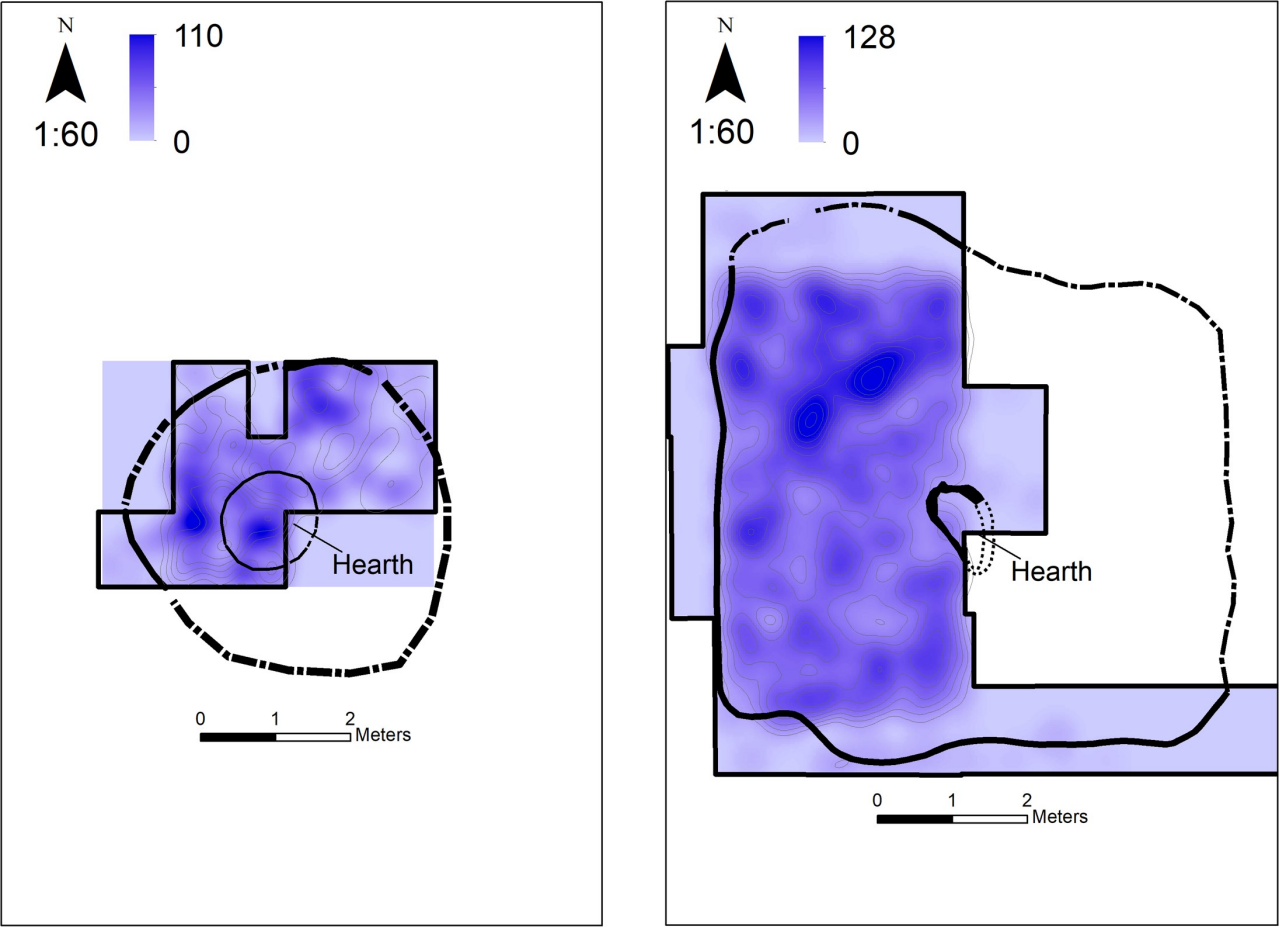
Excavated sites. Left: The earlier site - 12D56. Right: The later site - 12D16. The plans of the excavation sites showing the structures excavated (solid lines are places where the perimeter of the structure was excavated, dashed lines represent extrapolation of the structure parameter and are provided as context reference, shading is pottery density (sherd/m2), see S1 Text for details).

At the earlier site, 12D56 (also named Jiajiagou west site 贾家沟西遗址), the excavation exposed a single-occupation, irregular-oval structure of ~3.5 m in diameter, which contained ash, pottery and stone tools (Fig 3). Based on seven ^14^C ages, the site was occupied between 7.9–7.75 ka cal BP (Fig 2A; S1 Text, S3 Fig). This is the first time that a Xiaohexi phase site is radiocarbon dated. Previous research, based on ceramic typology, suggest dates in the 7^th^ and even 8^th^ millennium BC, but according to our findings the date of this phase cannot be earlier than 6,000 cal BC. The shape of the structure we excavated is also different from the supposedly rectangular structures reported for some Xiaohexi sites [34,35]. At some of those sites the rectangular structures may belong to a later Xinglongwa occupation [35,36] and in others the report is not detailed enough to examine the shape of the structures. Thus, it is possible that in other sites as well, structures were oval or that both oval and rectangular shape structure existed during this period.

**Fig 4:**
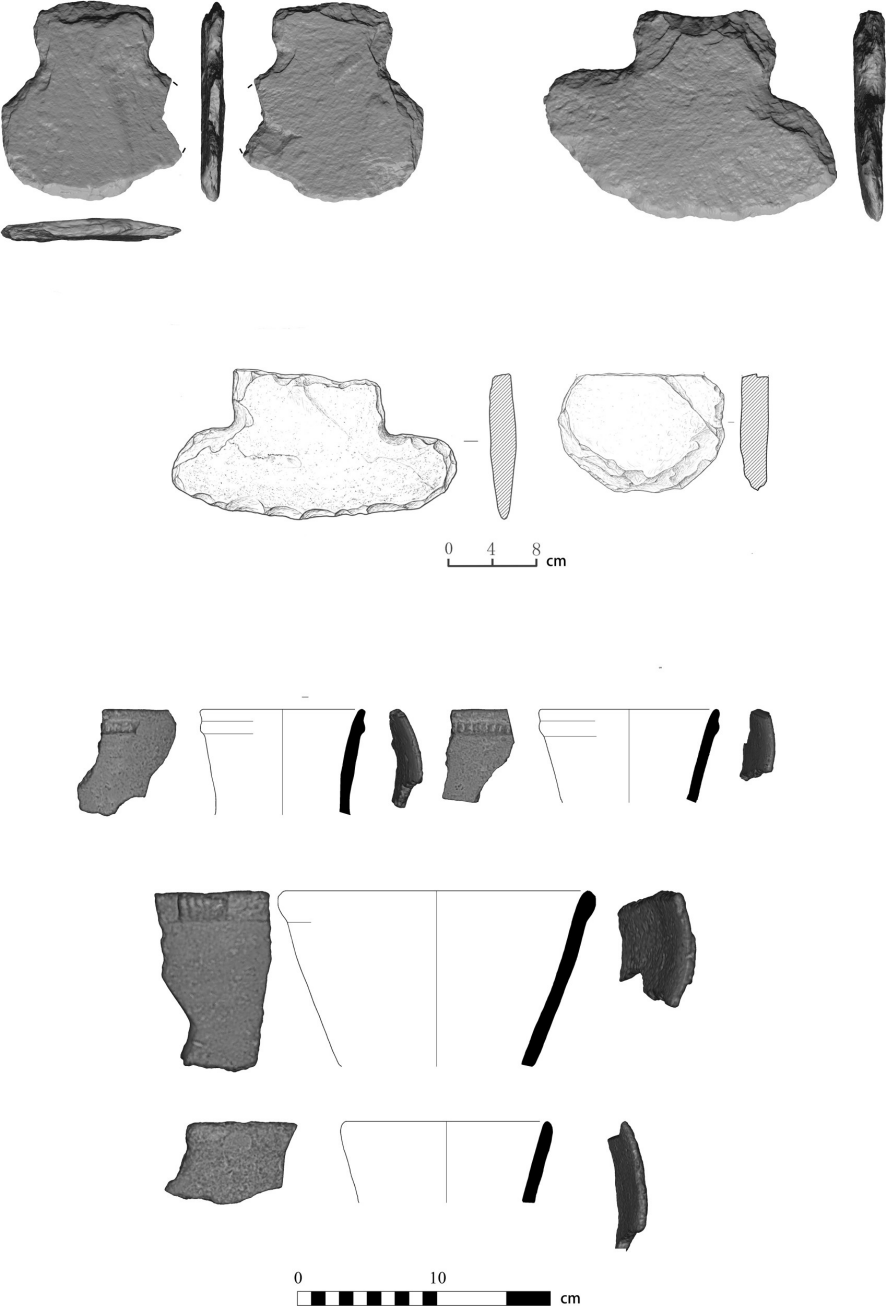
Artifacts excavated at site 12D56. Stone artifacts (upper half) and pot shards (lower half).

Potsherds (n = 300) excavated from this site are crude, soft and crumbly, similar in style and quality to those known from other Xiaohexi sites [34,37]. Most sherds are unadorned, but a few pots are decorated with appliqué and narrow bands of diagonal incisions (Fig 4). 330 stone artifacts were recovered, including ground and chipped stone tools. Of the ground stone tools, almost 80% are spades (S1 Text). Although no use-wear analysis was done, the fact that those artifacts, which are not known from earlier periods in the region, are so dominate suggest that they are associated with new set of activities, probably related to the clearance of woods and the cultivation of the land (Fig 4). Similar artifacts are known form other site dated to the Xiaohexi phase [34].

Floral remains from the site consist of a predominance of wild fruits and nuts and a small amount of wild Panicum millet (*Panicum miliaceum* subsp. *ruderale*). The two seeds that were recovered are small in size and are representative of what is expected of grains from early cultivated plants in the initial process of domestication (Supplementary Material).

At the later site, 12D16 (also named Tachiyingzi site 塔尺营子遗址), we excavated an area of ~40 m^2^ out of a much larger site. Based on ten ^14^C ages, the site was occupied between 7.5–7.4 ka cal BP (Fig 2A; S1 Text, S3 Fig). The area excavated contained a rectangular structure (house) measuring c. 7.5×6.5 m (area I, Fig 3) and remains of at least one additional structure (area H). The shape of the structure in area I is identical to those known from the nearby site of Chahi (查海), where a large area of the site was exposed reveling a densely occupied Xinglongwa period village [36].

**Fig 5:**
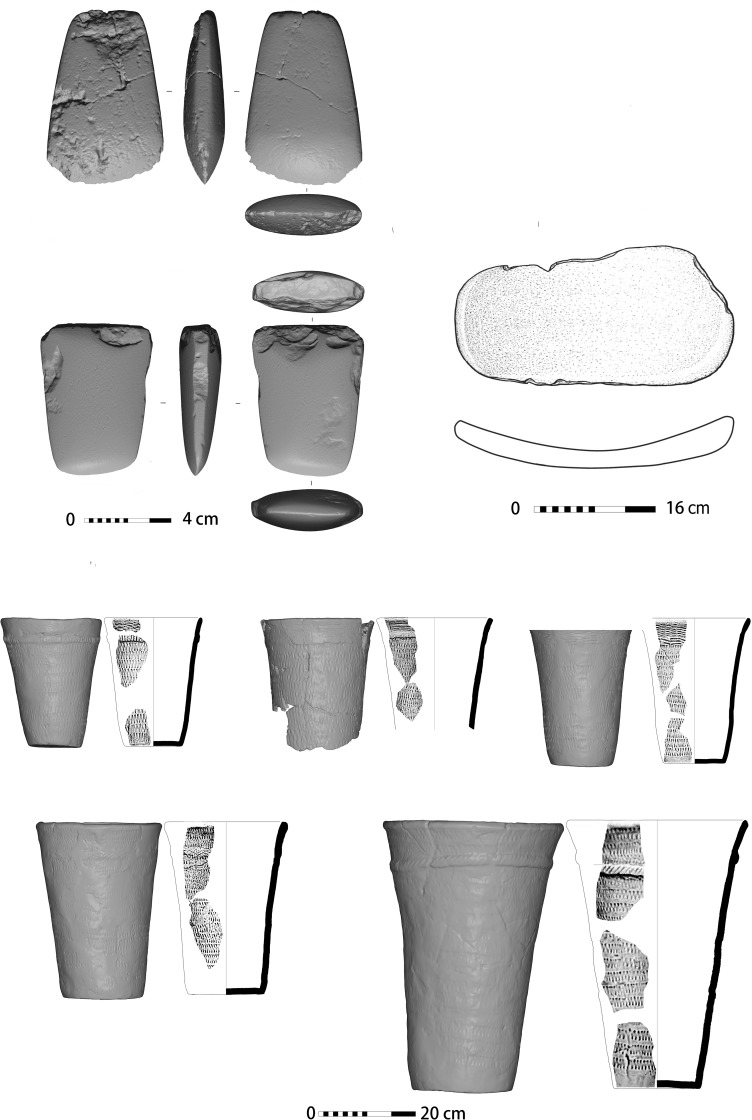
Artifacts excavated at site 12D16. Stone artifacts (upper half) and ceramic vessels (lower half).

The structure in area I was severely burned during or after its abandonment and remains found on its floor indicate that it was supported and covered with large oak beams. On its floor we recovered 22 complete ceramic vessels of typical Xinglongwa culture (Fig 5). The quality of ceramic production is high and most are highly decorated with incised motifs. The stone artifacts are more abundant and diverse (n = 1003 (than those of site 12D56, and show evidence for highly standardized pressure bladelets produced on-site, as a household routine. The ground stone assemblage consists of grinding stones, polished axes and adzes, spades, pounders and hammer-stones (Supplementary Material).

**Fig 6:**
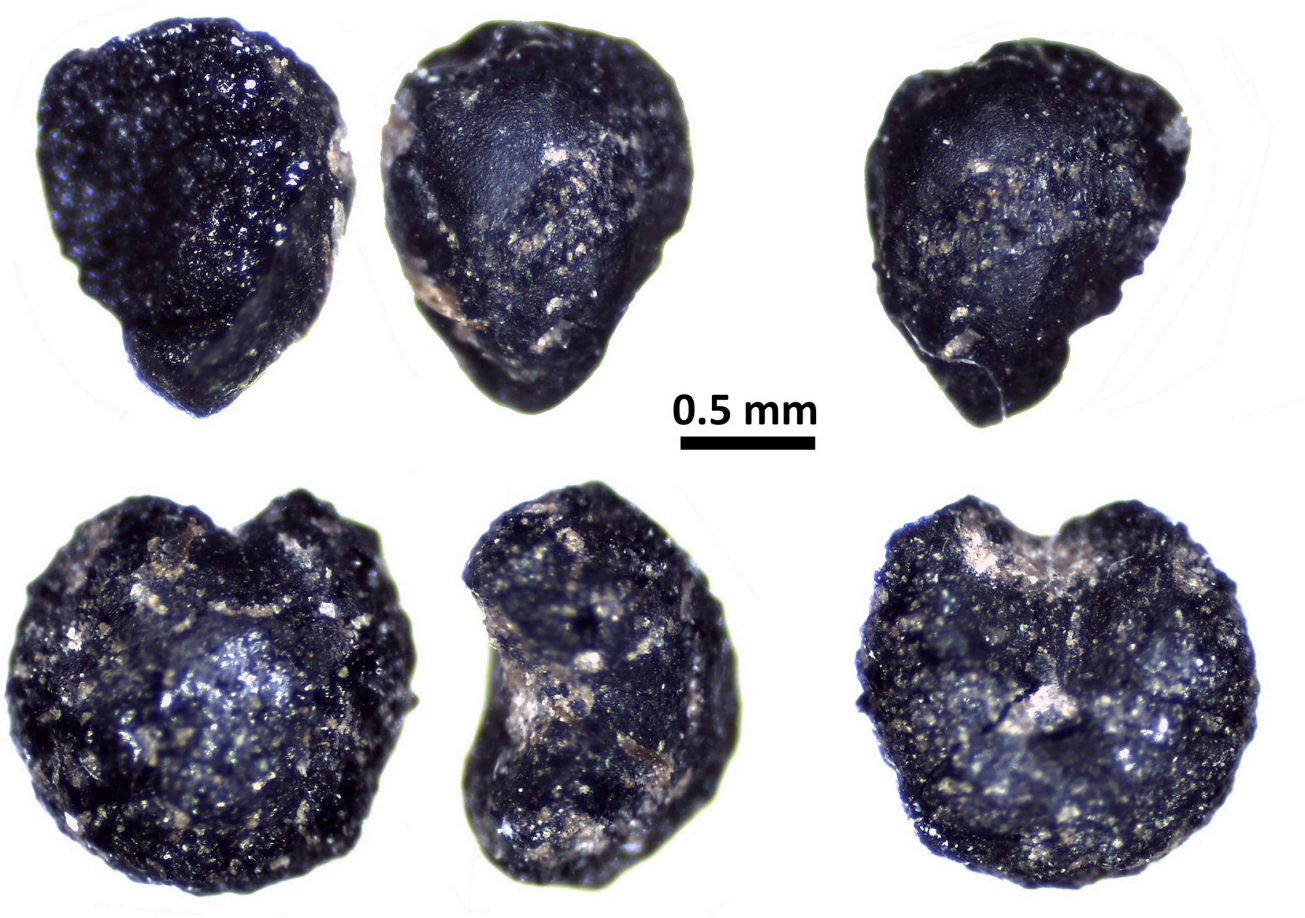
Remains of common millet (Panicum miliaceum) from site 12D16.

Floral remains include a few millet seeds but are predominantly composed of wild fruits and nuts. Sixteen millet grains are identified as common millet (*Panicum miliaceum*) (Fig 6). Their size is small, on average 1.25–1.70mm in length and 1.05–1.40 in width, closer to the wild type Panicum (*Panicum miliaceum* subsp. *ruderale*) than to modern domesticated broomcorn (Supplementary Material). These grains seem to represent plants that are not yet fully domesticated, but represent an early phase in a sequence of size change expected to take 2–3 ka [18].

### The climate record

To evaluate northeast China’s paleo-hydroclimate during the transition to domestication and sedentism, we integrated data from Dali lake-level [32], and Lianhua Cave oxygen isotopes [33](Fig 2D and 2E), which provide an estimate of rainfall change (former) and continuous, high-resolution absolute dating (latter). In addition, we analyzed soil δD_wax_ from both archaeological sites, which primarily records the isotopic composition of rainfall [38] (S1 Text). The paleoclimate records show similar hydroclimate patterns. Between 9–8.1 ka relatively dry conditions prevailed in northeast China. Between 8.1–7.9 ka a sharp substantial increase in rainfall amount occurred. The Lianhua record shows a 2.5‰ isotope depletion at 8.1 ka, which is only comparable in magnitude to the transition from the Younger Dryas to the Early Holocene at 11.5 ka. Soil δD_wax_ from the later 12D16 site is more depleted than that of the earlier 12D56 site and is thus in agreement with the other records (Supplementary Material) (Fig 2B). The relatively wet conditions prevailed for ~400 years. A decrease in rainfall amount occurred between 7.55–7.4 ka. This transition appears in both records, though the timing of the rainfall decline differs by ~150 years.

## Discussion

### Sedentism in Northeast China in the light of archeological theory

The onset of sedentism, cultivation and the early phase of domestication in northeast China is an important test case for studying the theoretical frameworks for the transition to agriculture. The transition to sedentism in the Xiaohexi phase is defined by investment in permanent structures, unknown in this region in prior periods, the dramatic increase in the production of pottery found at all sites in relatively large quantities, and the presence of large grinding stones. The empirical coincidence of the onset of sedentism and cultivation (at 7.9 ka) with the transition to a significantly wetter climate (at 8.1–7.9 ka) suggests that affluent conditions played an important role in facilitating the onset of these processes in northeast China. Our results suggest that in northeast China, fundamental and multi-scale changes occurred not due to necessity or stress but rather under stress-free affluent conditions and thus support the affluency hypothesis. Such plentiful resources allowed human communities to settle down in one place and enhance their interaction with their immediate environment, without the need to migrate in search of food.

The Xinglongwa culture thrived throughout this affluent period and declined within a century from the end of the wetter condition. This strengthens the notion that affluent climatic conditions were an important factor in the ability of this culture to thrive.

### The short path to sedentism and long path to agriculture

Sedentism and plant cultivation initiated simultaneously, but sedentism and the development of a complex village society matured much faster. The initial phase of sedentism, in the Xiaohexi phase is typified by small sites with makeshift irregular-shaped huts, which suggest low investment in construction and perhaps short life-use. This phase rapidly transitioned into the full-fledged Xinglongwa culture in which large scale-villages (~5 ha) containing up to 40 domestic structures, represent an increase in community size that coincided with a rapid increase of regional population levels. Rectangular domestic structures, like the one we excavated at site 12D16, and community-wide projects, such as the ditch that surrounded the Chahai site [36], suggest substantial investment, and communal construction efforts. Simultaneous processes, such as the intensification of craft production and improvement of ceramic and stone tool technologies, suggest a development of craft specialization and economic intensification. Similar developments are known from other parts of north China [25,39,40], suggesting that the processes we describe were shared by many contemporaneous societies.

The process of plant domestication progressed at a much slower pace. Elsewhere, researchers argue for an earlier date of millet domestication and use in other regions of North China [15,17,22,40]. However, because they use different types of data it is difficult to compare their results to ours. According to our study, evidence for its initiation (i.e. collection of plants that later will be domesticated and probably the cultivation of wild plants) is found at site 12D56, even after 500 years it was not yet completed at site 12D16. Moreover, the percentage of domesticated foods seems to have remained limited even during the height of the Xinglongwa period. Our results disagree with earlier studies that found human bone carbon isotopes from the Xinglongwa period suggestive of a C4 plant (possibly millet) dominated diet [41]. Future research will be needed to resolve these contradictions. Recent evidence from other parts of North China [40] suggest that like the process we describe for the Fuxin area, there too the trajectory of domestication and transition to agriculture was relatively long. In the initial phases, addressed by this paper, it was not domestication *per-se* which was important but the cultivation of the land (indicated by the large number of spades found at site 12D56) and the harvesting and consumption of durable food resources such as seeds (of domesticated and wild plants), nuts (such as acorn and walnut) and wild fruits (apricot, Amur cork). Those resources were collected in large quantities, stored for long periods, and thus enable a year-long occupation of the same site and the stable support of larger communities. Based on the data we collected, we suggest that the suite of traits that we find in the earliest phases–sedentism, expansion into new types of food resources and perhaps cultivation—were the driving force behind domestication and not *vice-versa*.

## Conclusions

The archaeological, botanical and paleo-climatic data presented above help clarify the context of the transition to a sedentary way of life, plant cultivation and initial stages of plant domestication in Northeast China. The multi-dimensional nature of our data allow us to suggest that these processes occurred during a period of comparative affluence that enabled population increase and experimentation with new resources and technologies. Our botanical findings support the view that the domestication process took a long time after humans started cultivating plants, as has been demonstrated for several other crops in other regions [18] and that not all the plants that were initially cultivated or intensively collected were botanically transformed and became domesticated. These observations, which may not be true for all cases of independent transitions to sedentary agricultural societies, are crucial for a global view of the evolution of human society. They suggest that at least in some cases, fundamental and multi-scale changes occurred not due to necessity or under stress but rather because of opportunities to experiment and expand under stress-free affluent conditions.

## Supporting information

S1 Fig Normalized population density averaged from both survey regions. (TIF)

S2 Fig Radiocarbon ages from both sites. Vertical error bars represent the 1σ of the calibrated age.
(TIF)

S3 Fig δD_wax_ sampling location in Area A of site 12D56. The structure was dug into natural soil that sits on top of granite bedrock. The samples (A1-A3) were sampled in the natural soil, archeological material and the modern topsoil.
(TIF)

S4 Fig δD_wax_ sampling location in Area H. The top figure shows a profile of a structure dug into the natural soil, which is filled with archaeological material. The bottom figure shows a close-up of the sampling locations of δD_wax_ (H1, H3, H5 and H8) and results of radiocarbon samples as a function of depth.
(TIF)

S5 Fig δD_wax_ sampling location in Area I. The sample (I-3) was sampled in the burnt layer associated with the broken vessels lying on the floor of the structure.
(TIF)

S6 Fig δD_wax_ results from the two sites. The earlier site (12D56) is in purple; the two structures from the later period are in orange (area H) and blue (area I). The samples that predate the site were assigned an arbitrary age of 9500 BP to prevent confusion, but the only age constraint we have is that they are older than the archaeological occupation.
(TIF)

S1 Text (DOCX)
