# Supplementary figures and images for: Sedentism and plant cultivation in northeast China emerged during affluent conditions
Source: PLoS One. 2019 Jul 18;14(7):e0218751. doi: 10.1371/journal.pone.0218751 (PMC6638895; doi:10.1371/journal.pone.0218751)

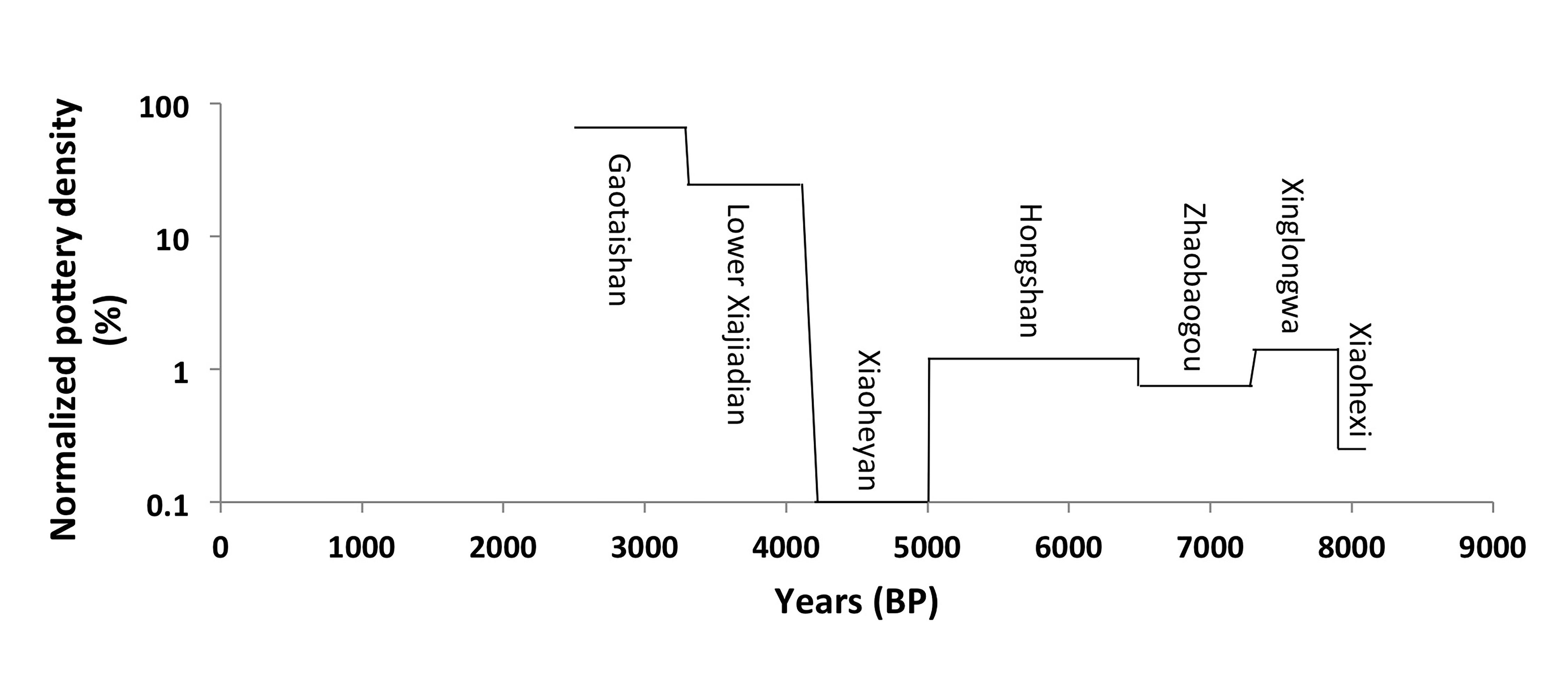

Supplement: S1 Fig — (TIF) [file pone.0218751.s001.tif]

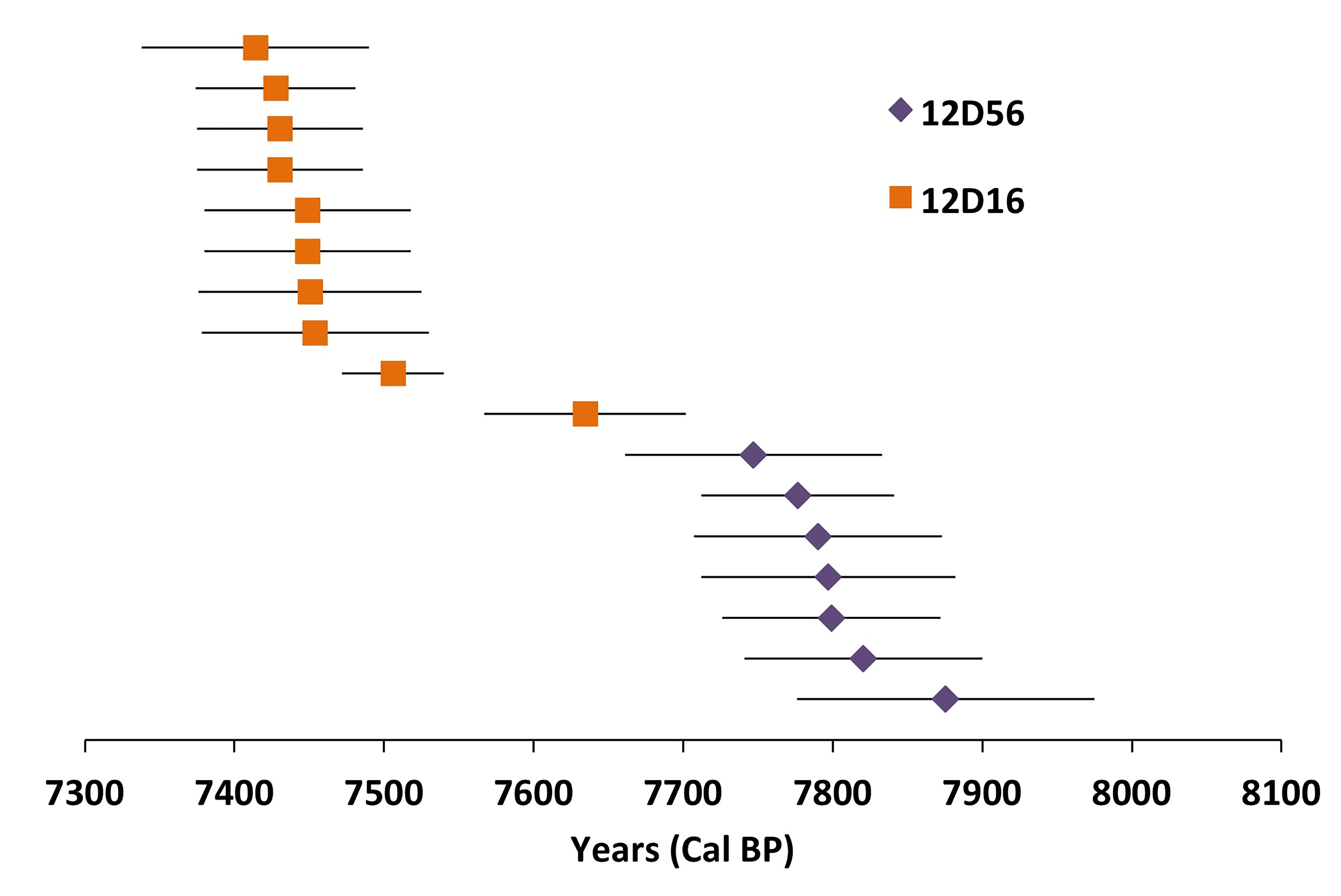

Supplement: S2 Fig — Vertical error bars represent the 1σ of the calibrated age. (TIF) [file pone.0218751.s002.tif]

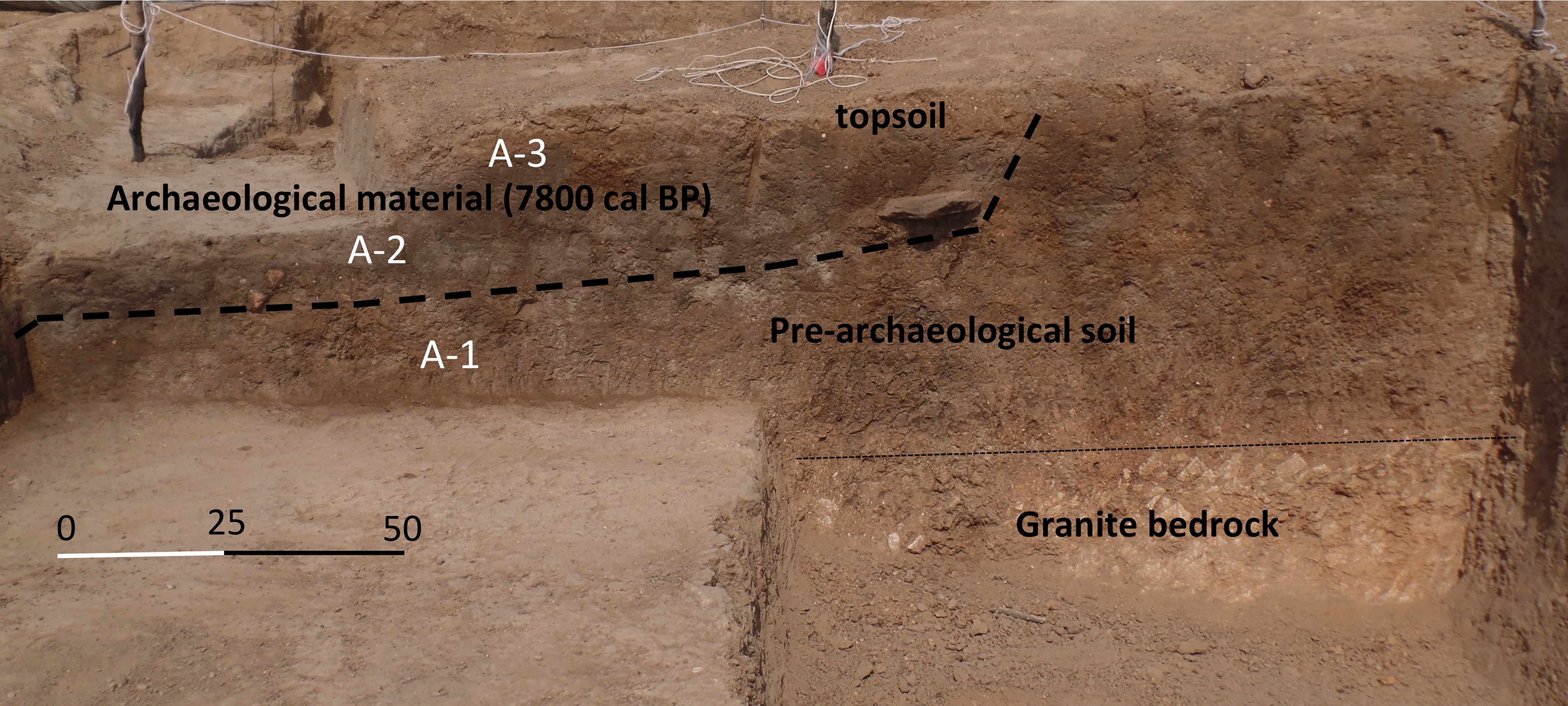

Supplement: S3 Fig — The structure was dug into natural soil that sits on top of granite bedrock. The samples (A1-A3) were sampled in the natural soil, archeological material and the modern topsoil. (TIF) [file pone.0218751.s003.tif]

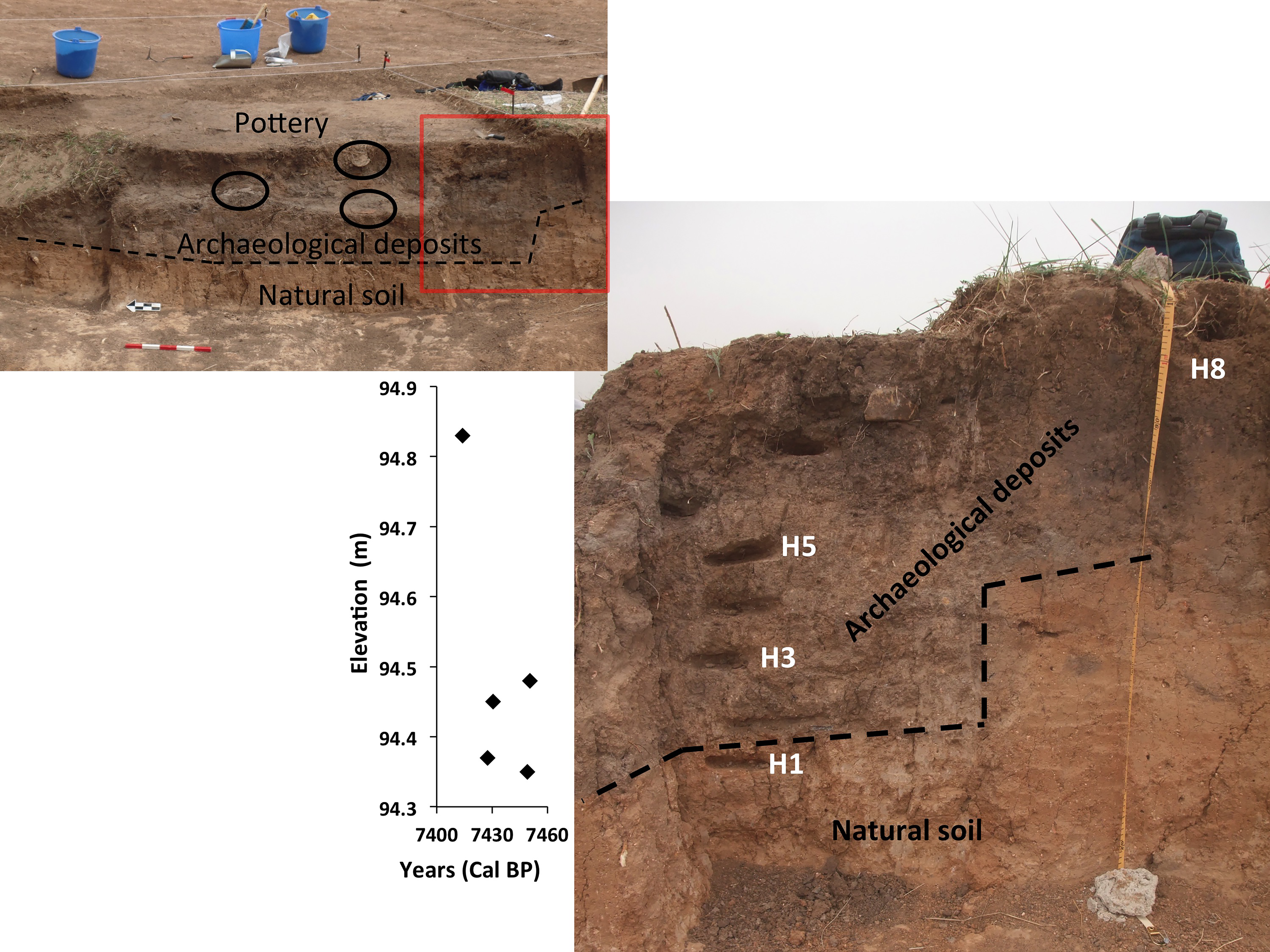

Supplement: S4 Fig — The top figure shows a profile of a structure dug into the natural soil, which is filled with archaeological material. The bottom figure shows a close-up of the sampling locations of δDwax (H1, H3, H5 and H8) and results of radiocarbon samples as a function of depth. (TIF) [file pone.0218751.s004.tif]

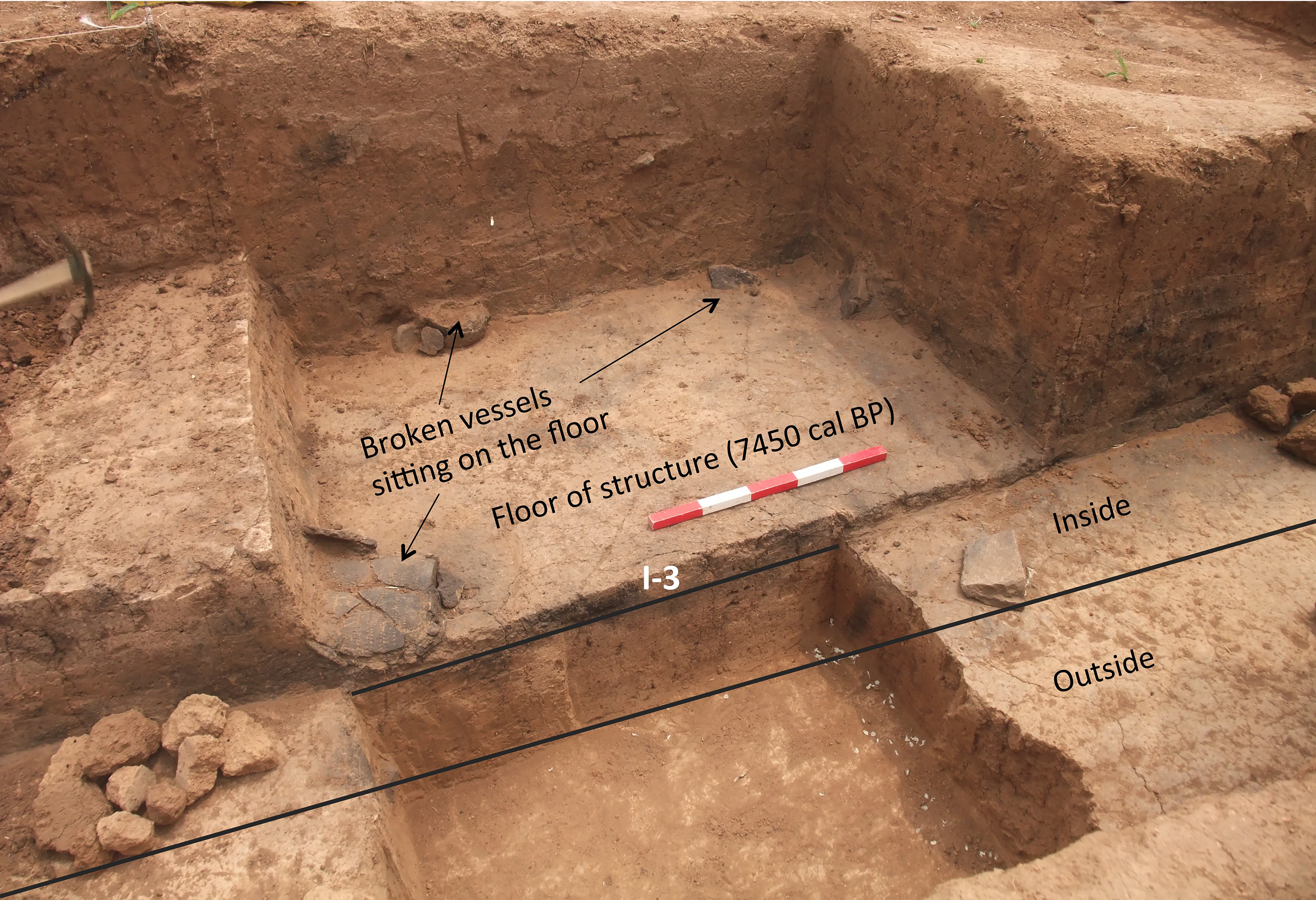

Supplement: S5 Fig — The sample (I-3) was sampled in the burnt layer associated with the broken vessels lying on the floor of the structure. (TIF) [file pone.0218751.s005.tif]

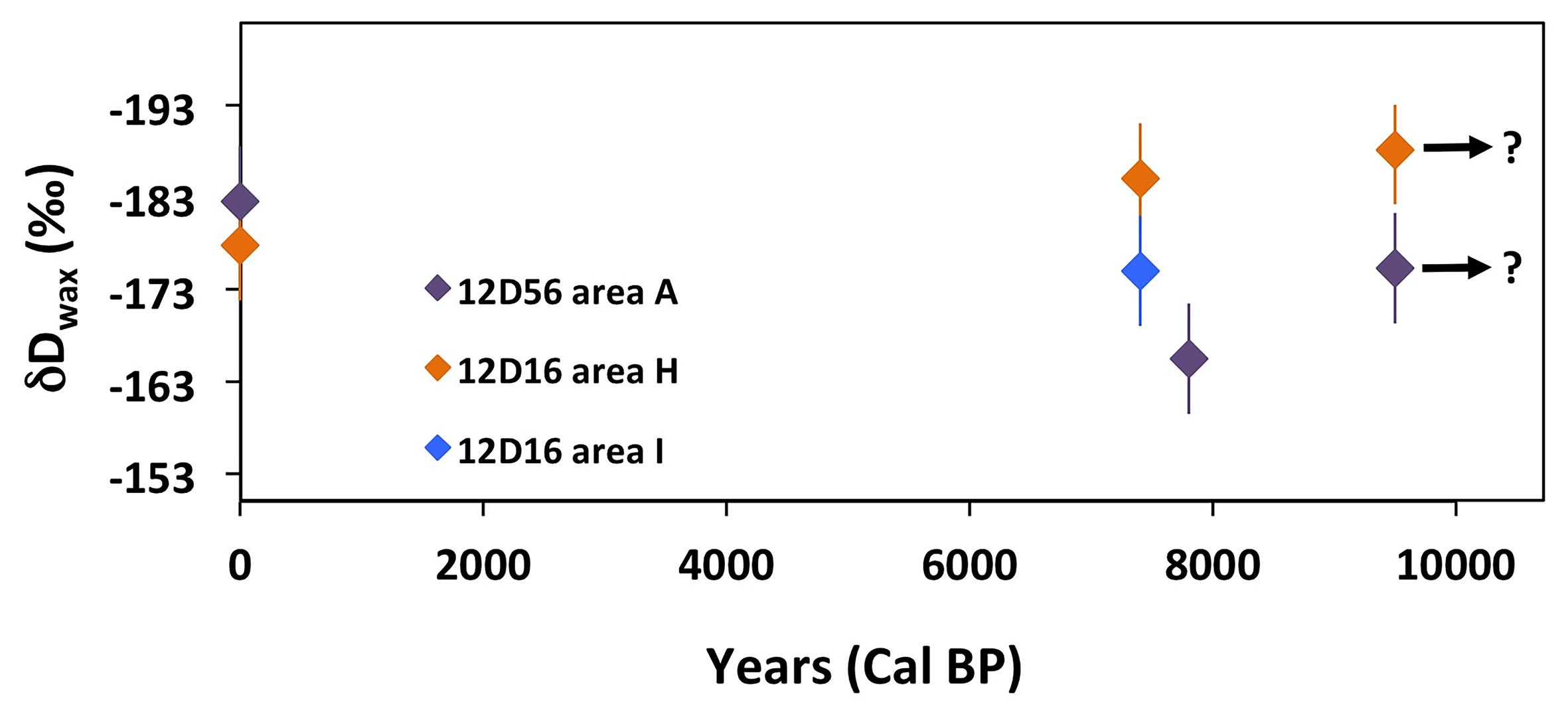

Supplement: S6 Fig — The earlier site (12D56) is in purple; the two structures from the later period are in orange (area H) and blue (area I). The samples that predate the site were assigned an arbitrary age of 9500 BP to prevent confusion, but the only age constraint we have is that they are older than the archaeological occupation. (TIF) [file pone.0218751.s006.tif]
