# Supplementary material for: Sedentism and plant cultivation in northeast China emerged during affluent conditions
Source: PLoS One. 2019 Jul 18;14(7):e0218751. doi: 10.1371/journal.pone.0218751 (PMC6638895; doi:10.1371/journal.pone.0218751)
Supplement: S1 Text — (DOCX) [file pone.0218751.s007.docx]

Supplementary material for:

**Sedentism and plant cultivation in northeast China emerged during affluent conditions**

Gideon Shelach-Lavi^1,2*^, Mingyu Teng^2^, Yonaton Goldsmith^3,11*^, Ido Wachtel^4^, Chris J. Stevens^5^, Ofer Marder^6^, Xiongfei Wan^7^ Xiaohong Wu^8^, Dongdong Tu^1^, Roi Shavit^6^, Pratigya Polissar^9^, Hai Xu^10^, Dorian Q Fuller^5^

^1^Department of Asian Studies, The Hebrew University of Jerusalem, Jerusalem, Israel.

^2^Center for Frontier Archaeology, Jilin University, Changchun, China.

^3^Department of Geological and Planetary Sciences, California Institute of Technology, Pasadena, CA 91125, USA.

^4^ Department of Archaeology, The Hebrew University of Jerusalem, Jerusalem, Israel.

^5^Institute of Archaeology, University College London, London, England.

**^6^** Department **of Bible Studies, Archeology and the Ancient Near East**, Ben-Gurion University, Beer Sheva, Israel.

^7^Institute of Archaeology and Cultural Relics, Liaoning Province, Shanyang, China.

^8^School of Archaeology and Museology, Peking University, Beijing, China.

^9^Lamont Doherty Earth Observatory, Palisades, NY 10964, USA.

^10^Institute of Surface-Earth System Science, Tianjin University, Tianjin 300072, China.

^11^Institute of Earth Sciences, The Hebrew University of Jerusalem, Jerusalem, Israel.

^*^Corresponding Authors: [gideon.shelach@mail.huji.ac.il](mailto:Gideon.shelach@mail.huji.ac.il) (GS); [yonig@caltech.edu](mailto:yonig@caltech.edu) (YG)

**Supplementary Materials 1**

**S1a: Survey method and Methods of population density estimation**

Our population density estimates are based on the results of two systematic regional surveys conducted in Northeast China: the Chifeng and the Fuxin surveys [27,28]. These surveys covered 1,234 km^2^ at 50 m resolution and 105 km^2^ at 20 m resolution, respectively, i.e. a total of 1,339 km^2^ was traversed over 11 field seasons. The raw data of those two surveys are deposited and open to the public in the Comparative Archaeology Database, University of Pittsburgh. URL: <http://www.cadb.pitt.edu>. Fuxin survey data: http://www.cadb.pitt.edu/shelach/index.html

Chifeng survey data: http://www.cadb.pitt.edu/chifeng/index.html

The methodology of both surveys is similar and is based on defining a collection unit – rather than a ‘site’ – as the analytic unit for calculating the density of artifacts. The survey team was spread out along a line (at 20 or 50 m apart) perpendicular to the walking direction. Once the survey team identified artifacts (potshards or stone tools) it opened a collection unit. Each unit was no more than 100x100 m, and whenever the area covered by artifacts was larger, additional collection units were opened until an area with no pottery was reached. The boundaries of each collection unit were documented using GPS and then transferred as polygons to our GIS system (using ArcGIS software). All pottery and stone tools were counted, identified and ascribed to an archaeological period and linked to a database containing the physical parameters of the collection units (area, location etc.).

Within the borders of the collection unit we marked a 3 m diameter circle (7.1 m^2^) arbitrarily and all artifacts found within were collected (with up to three such circles per unit, to allow for a large enough sample size). A collection unit in which the collection circle contained more than 3 shards was defined as a ‘systematic collection unit’ (SC) and the artifact density in the circles was assumed to represent the entire collection unit. If the artifacts were so sparse that a 3 m diameter circle produced less than three artifacts, then the unit was defined as a ‘general collection unit’ (GC).

Wherever systematic collections were conducted, pottery density (q_S_) was derived by dividing the number of shards from each period recovered within the collection circles (P_c_) by the area of the circle (or circles) (A_c_):

$$q_{S}=\frac{P_{c}}{A_{c}}$$

For example, a circle 3 m in diameter has an area of 7.1 m^2^, so, if two collection circles were initiated and 10 Xinglongwa shards were found in them, then the collection unit would be taken to have a shard density of 0.7 shards per m^2^ for the Xinglongwa period (10 shards / 14.2 m^2^= 0.7 shards per m^2^). If, in addition, 6 Gaotaishan shards were found, the collection unit would be taken to have a shard density of 0.42 shards per m^2^ for the Gaotaishan period (6 shards / 14.2 m^2^= 0.42 shards per m^2^).

Calculation of surface artifact density values for general collection units was more complicated than for systematic collections, because we know that even a relatively intensive survey cannot recover all the shards scattered in an area that is c. 1 ha in size. The calculation is:

$$q_{G}=\frac{P_{cu}}{A_{cu}}xC$$

Where q_G_ is the pottery density per archeological period in the general collection unit, P_cu_ is the amount of pottery per archaeological period in the collection unit, A_cu_ is the area (m^2^) of the collection unit and C is a dimensionless constant, which we defined as 100 (see below).

As explained above, the decision to carry out a general collection rather than a systematic one means that surface ceramic densities were subjectively estimated to be low enough that a systematic collection circle would not yield as many as three or four shards. This threshold is equivalent to about 0.5 shards per m^2^ (3.5 shards/7.1 m^2^ = 0.5 shards per m^2^). Our experience has shown that even the largest general collections usually produce no more than 50 shards, and even such large collections, if divided by the area of the collection unit, result in very low density estimates. For example, a collection of 30 shards from a collection unit of 1 ha (10,000 m^2^) would produce an estimate of 0.003 shards per m^2^. Assuming that this collection method yields a shard density that is below, but not much below, the threshold of conducting systematic collection, we looked for a constant number by which we should multiply the result of the direct calculation to get more reliable density estimates. After experimenting with different constants, we arrived at the estimate that multiplying by 100 would produce such results. Again using the hypothetical example, multiplying the calculated shard density by 100 gives an estimate of 0.3 shards per m^2^, still lower than the systematic collection threshold, but representing a high value for a general collection. Collections where fewer shards were collected will produce even lower values. These estimates are less accurate than estimates based on the results of systematic collections but, nonetheless, they provide us with rough density figures for collection units in which we already know that the density of shards was low (*2-4*).

To calculate a pottery amount for each period (P_Tot_), we multiplied the estimated shard densities (q_GC_ and q_SC_) by the area of the collection units (A_cu­_) for both the systematic and general collection units and summed. We assume that the amount of discarded pottery is also a function of the length of the archaeological period (i.e. the longer the period the more pottery is discarded); therefore, we normalized the pottery amount to the length of the period (T_period_).

$$P_{Tot}=\frac{\sum\left( q_{SC}xA_{cu} \right)+\sum\left( q_{GC}xA_{cu} \right)}{T_{period}}$$

**Combining the results of both surveys.** As we have two survey regions, we combined the normalized pottery amount from both areas in two ways, a) by weighing the results by area (i.e. summing the results from both areas) and b) by giving equal weight to both areas (i.e. averaging the results from both areas). The results of the long-term trend of both methods are similar. In figures 2 and S1 Fig. we plot the sum of both areas.

**Scaling pottery amount to population density.** We hypothesize that the pottery amount found on the landscape during a systematic archaeological survey scales with population density [31]. Thus our calculation of scaled pottery amount per period (Fig 2 and S1 Fig) scales with population density through time and provides a means to assess how population density changed through time in the survey regions.

**S1 Fig. Normalized population density averaged from both survey regions.**

**S1b: Excavation methods**

We selected for excavation two single-occupation sites discovered during the Fuxin regional survey (Fig 1). The first (site 12D56) contained pottery shards identified as the earliest phase of the Xinglongwa culture (also known as the Xiaohexi culture). The second (site 12D16) contained pottery shards identified as typical Xinglongwa culture. All the raw data of the excavations of those two sites are deposited and open to the public in the Comparative Archaeology Database, University of Pittsburgh. URL: http://www.cadb.pitt.edu/shelach/index.html.

The sites were excavated at a 1 m^2^ grid and 5 cm vertical resolution. All the excavated earth from the anthropogenic strata was screened through a 2 mm sieve and part was wet-screened. The locations of artifacts, features, samples, etc. were documented using a Total-Station. Samples for flotation (through 280 micro mesh) and radiocarbon dating were systematically collected for each grid square, and further analyzed in the lab.

**S1c: Radiocarbon ages from the archaeological sites**

To assess the ages of both sites, seven and ten radiocarbon samples were sampled in the early (12D56) and late (12D16) sites, respectively (S1 Table). During the excavation, whenever visible charcoal was identified, it was collected into tin foil using a clean trowel to prevent contamination. In site 12D56, the seven samples were collected from the occupation horizon in and around the hearth in the center of the structure. In area H of site 12D16, charcoal was collected throughout the section. In area I of site 12D16, charcoal was sampled at the living floor stratum throughout the excavated structure.


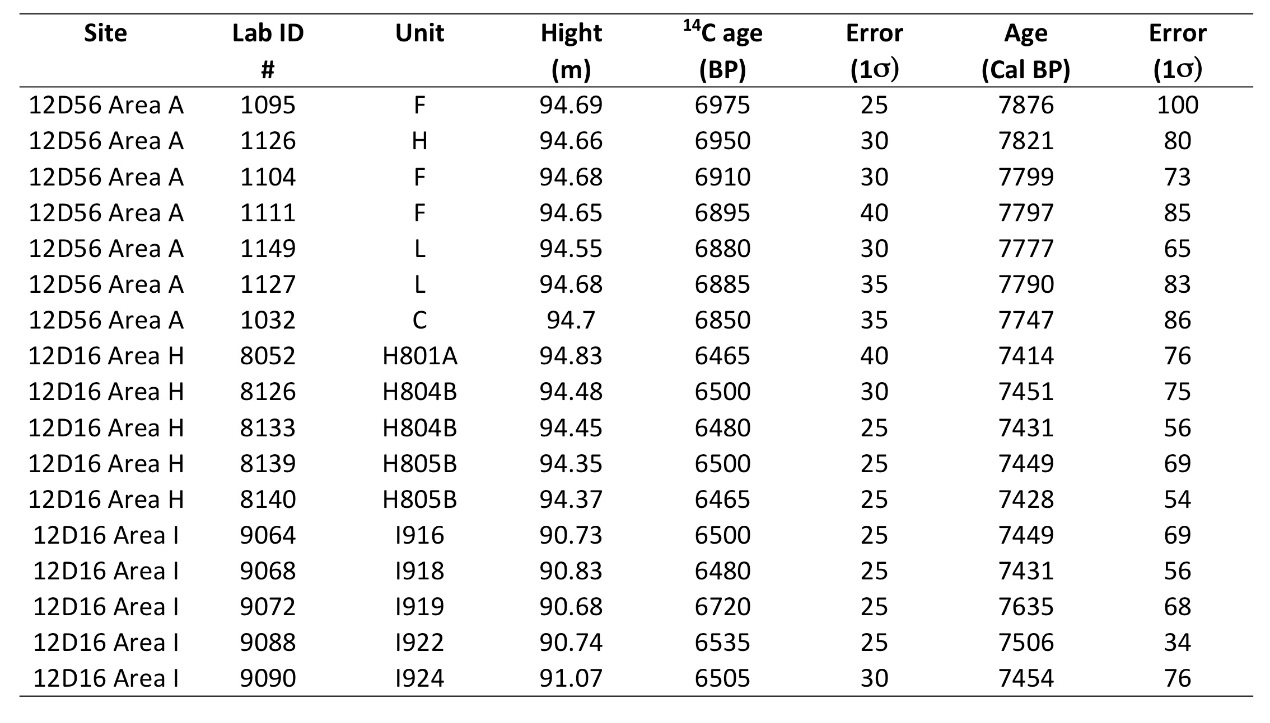


**S1 Table. Radiocarbon data.**

Radiocarbon measurements were conducted at the 14C lab of the School of Archaeology and Museology, Peking University, following conventional procedures. Protocol for sample pretreatment and preparation is: All charcoal samples were observed under microscope and the fabric roots and other impurities were picked out. After supersonic cleaning, the samples were treated by the normal AAA procedures. Samples were weighted and sealed with copper oxide and silver in quartz tubes under a vacuum system. The combustion temperature was 850℃. The CO_2_ from the tubes was purified and transferred into the gas container separately. The reduction from CO_2_ to graphite was performed with H_2_/Fe in the vacuum graphitization system. The Fe catalyst has to be cleaned and activated under 450℃ with O_2_ and H_2_ separately before reduction. The graphite was formed at 540℃. AMS radiocarbon measurements were done at the School of Physics, Peking University. The AMS system is based on a National Electrostatics Corp. (NEC) 1.5SDH-1 0.5MV pelletron with 40-sample MC-SNICS ion source. The accuracy of this system is better than 0.4% and the machine background is lower than 0.03pMC.

A ^14^C half-life of 5568 years was used and aged are assgined as BP from 1950 years of age. Calibration was done using OxCal online v4.2.4 [42] and IntCal 13 [43] (S2 Fig).

**S2 Fig. Radiocarbon ages from both sites. Vertical error bars represent the 1σ of the calibrated age.**

**S1d. Excavated pottery density method**

Assessing the pottery and stone tool density in the excavated sites was achieved as follows: a) During the excavation, the location of large (>2 cm) artifacts was determined by Total- Station measurements; b) Artifacts found during sieving were assigned a random coordinate (using the ‘rand’ function in Excel) within the square (1x1 m) and spit (typically 5 cm). The counters were calculated using the ‘Nearest neighbor’ function in the ArcGis program.

**S1e: Plant remains and radiocarbon dates from excavations of Xinglongwa Period sites, Fuxin County, Liaoning**

Plant remains were analyzed from 90 samples collected during the 2015 excavations of the two Xinglongwa cultural sites: Site 12D56 (n=19) and Site 12D16: Area H (n=14), Area I (n=54) and Area C (n=3). This dataset is the second archaeobotanical dataset for Xinglongwa Culture sites within northeast China and the only one for which there is a good chronological control for early and mature Xinglongwa strata. The range of material seen within the two sites was broadly similar, but some differences were noted.

Site 12D56 represents the oldest phase of occupation with dates on wood charcoal at 2-sigma ranging between 5980-5660 cal. BCE. Fruit stones and nut fragments are the most common plant remains at the site. Three possible seeds of more morphologically wild type Panicum millet (*Panicum miliaceum* subsp. *ruderale* (Kitag.) Tzvelev) were recovered. In addition, seeds and fruit of Amur cork, *huang bo shu* (黄檗属; *Phellodendron amurense* Rupr.), were recovered. A radiocarbon age on a fruit fragment gave an age of 5990-5790 cal. BCE (S2 Table).

| **Lab**  **Code** | **Material**  **(all charred)** | **Site** | **Context** | **δ^13^C‰** | **Radiocarbon**  **(BP)** | **Calibrated**  **date (95.4%)** |
| --- | --- | --- | --- | --- | --- | --- |
| SUERC-  3818 | *Phellodenron*  *amurense* (fruit fragments) | 12D56 | 1094 | -25.2 | 6997±30 | 5990-5790  cal. BCE |
| SUERC-  3816 | *Crataegus*  *pinnatifida* stone | 12D16 | Area C  304 3008 | -24.4 | 6607±30 | 5620-5480  cal. BCE |
| SUERC-  73817 | *Juglans*  *mandshurica* | 12D16 | Area I  943 9139 | -24.2 | 6600±30 | 5620-5480  cal. BCE |
| SUERC-  3819 | Nut fragments,  cf. *Nelumbo nucifera* | 12D16 | Area H  805 8142 | -24.2 | 2508±29 | 790-540  cal. BCE |
| Beta-  471172 | *Armeniaca* sp. | 12D16 | Area H  805 8142 | -24.1 | 6460±30 | 5490-5360  cal. BC |

**S2 Table: Radiocarbon data on seeds**

The younger site 12D16 (ranging between 5530 to 5330 cal. BCE and 5710 to 5370 cal. BCE, respectively) contains nut fragments of walnut (*Juglans* cf. *mandshurica* Maxim.), apricot stone (*Armeniaca* sp.), a lotus seed (*Nelumbo nucifera* Gaertn.), seeds of the Amur cork fruit, porcelain-berry or *she-putao* (*Ampelopsis glandulosa* (Wall.) Momiy. or *A. delavayana* Planch. Ex Franch.) and a seed from the Rosaceae family. Fourteen millet grains were recovered, nine of which are small *Panicum miliaceum* L., which are more similar in size to wild type Panicum (*Panicum miliaceum* subsp. *ruderale* (Kitag.) Tzvelev) and five of which are Setaria millet (*Setaria italica* (L.) P. Beauv., *Setaria viridis* (L.) P. Beauv. and *Setaria* sp.). Radiocarbon determinations on a fruit stone (area C), walnut (area I), and apricot (area H) gave ages of 5620 – 5360 cal. BC (S2 Table).

The grains of broomcorn millet (*Panicum miliaceum* L.) recovered from the younger site are of high significance in that they are representative of what is expected of grains from early cultivated plants undergoing domestication. While the single grain of *Setaria italica* (L.) P. Beauv. appears fully domesticated but is suspected to be modern/ intrusive, the presence of seeds of *Setaria viridis* (L.) P. Beauv. along with *Echinochloa* sp. is in accordance with a scenario in which early cultivated fields comprised a mix of wild-type early cultivars and wild grasses.

The evidence from this site is consistent with evidence of early cultivation for other cereals, rice in the Lower Yangtze, and emmer, barley and einkorn in the Near East. In all these cases grains under pre-domestication cultivation, or the early stages of the domestication process, are morphologically similar in size and shape to those of the wild progenitors [18]. As seen in these sites, wild foods appear more important in these earliest stages of domestication than cereal grains for peoples that first undertook the cultivation of these cereals, with a gradual transition to an increased reliance on cereals and other cultigens during the middle to later Neolithic [44,45]. While sedentism appears already established in the Near East prior to the first cultivation of cereals, in China, the earliest appearance of cultivation tools appears to accompany increased evidence for sedentism and this would also appear to be similar for millets in Northeast China where hoes and house structures are present on the earliest Xinglongwa sites [19].

**Methods**

15-20 litre soil samples were collected from the various areas of the two sites. The locations of the samples were recorded by a Total-Station and entered into the excavation database. The samples were processed using standard bucket flotation using a 0.3mm mesh to retain the flot. The heavy fraction remaining within the bucket was laid out and dried and checked for charred remains and charcoal visible to the naked eye, pottery and lithics (no animal bone survived on the site) and then discarded. Flots were dried within the mesh bags in which they were collected. These were then transferred to plastic bags for transport when they were fully dried. The flots were sieved and each fraction sorted within the archaeobotany lab at Peking University, Beijing, and within the University of London. Charred material was extracted and identified where possible to genus or species level, with references to seed atlases [46–50], and modern reference material held at the Institute of Archaeology, UCL, London. Samples were then tabulated within Tables 1-4**,** following the nomenclature as given in *The Flora of China* [51].

Four initial samples were sent for radiocarbon dating at SUERC (Scottish Universities Environmental Research Centre), Glasgow, from each of the four areas. In light of one of the results, a further sample from 12D16 Area H was sent for radiocarbon dating.

**Results**

Site 12D56

Fruit stones, seeds, and nut fragments are the most common plant remains in site 12D56 (S3 Table). These comprised apricot stones (*Armeniaca* sp., ), Amur Cork (*Phellodendron amurense* Rupr) and occasional fragments of walnut (*Juglans* cf. *mandshurica* Maxim.). Amur Cork is found wild within Liaoning today [52], but has long been cultivated, predominately for its bark, which is used in traditional Chinese medicine. Fragments of apricot were thinner with a rough, scabrous outer surface, sometimes with a faint reticulate pattern, or lightly pitted. In some specimens, traces of the triple ridges or keels running alongside the ventral suture were also noted. Some of the fruit fragments from 12D56 104F were sent for radiocarbon dating.

| **Species** | **科/属/类** | **Ubiquity n** | **Ubiquity %** | **No. of Seeds** |
| --- | --- | --- | --- | --- |
| *Juglans mandshurica* (frgs) | 胡桃楸 *hu tao qiu* | 5 | 26.32 | 4 |
| *Kochia prostrata*/*scoparia* | 木地肤/地肤 | 1 | 5.26 | 1 |
| *Nelumbo nucifera* | 莲 *lian* | 2 | 10.53 | 2 |
| *Euryale ferox* | 芡实 *qian shi* | 1 | 5.26 | 0 |
| *Craetagus* sp. (thorn) | 山楂属 | 1 | 5.26 | 1 |
| *Armeniaca vulgaris* / *sibirica*/ *mandshurica* stone | 杏/山杏/东北杏 | 8 | 42.11 | 14 |
| *Phellodendron amurense* (seeds) | 黄檗 (种子) | 7 | 36.84 | 1 |
| *Phellodendron amurense* (fruit) | 黄檗 (果和) | 5 | 26.32 | 10 |
| cf. *Diarthron linifolium* | 草瑞香 | 3 | 15.79 | 5 |
| Onagraceae/Boraginaceae. Small seeds indet. | /紫草科 ( (未鉴定的种) | 1 | 5.26 | 2 |
| Culm node (Poaceae) | 秆节点 (禾本科) | 3 | 15.79 | 4 |
| Paniceae indet. | 黍族 | 2 | 10.53 | 0 |
| *Panicum ruderale*/*miliaceum* | 野生稷/稷 ((黍)) | 1 | 5.26 | 0 |
| Small seed indet. | 小种子 (未鉴定的种) | 1 | 5.26 | 0 |
| Seeds indet. | 种子 (未鉴定的种) | 5 | 26.32 | 5 |
| Nut fragments | 简而言之 | 2 | 10.53 | 22 |
| Fruit/Nut fragments indet. | 核 /简而言之 (未鉴定的种) | 11 | 57.89 | 84 |

**S3 Table: Flotation results of site 12D56**

Two possible grains of small wild type *Panicum* millet (*Panicum miliaceum* subsp. *ruderale* (Kitag.) Tzvelev) were recovered and one immature grain.

A single seed of *Kochia prostrata/scoparia* came from Site 12D56 (Unit 102L, sample 1147). The plant generally grows as a weed, but *Kochia* *scoparia* (L.) Schrad. is also consumed as a green vegetable.

A broken half achene of probable *Humulus scandens* (Lour.) Merr. (葎草属 *lü cao shu*) was recovered from 12D56, 102A (1086). Two small unidentified Boraginaceae-type seeds were recovered from unit 104F, sample 1094.

Five undefined seeds of the same kind were recovered from the site, each around 2.3mm long and just over 1mm wide. The seed is raindrop or tear shaped, being smooth and rounded at one end coming to a sharp point with a distinctive possible hilum or embryo/radical scar at the other end. The general shape and size would suggest that they are most likely within the Thymelaeaceae, closely resembling those of *Diarthron vesiculosum* (Fisch. & C.A.Mey.) C.A.Mey., although in China this species is recorded only in Xingjiang [51]. Of the 9 genera endemic in China most are distributed in southern and western China and only a few species, e.g. *Daphne*, *Diarthron* and *Stellera*, grow in northeast China. From the *Flora of China* [51], the most likely candidate would be *Diarthron linifolium*, which has similar sized seeds, and while it is not recorded in Liaoning, it is recorded in neighbouring Jilin and Hebei, as well as in Shanxi and Mongolia. The plant is predominately a weed, and while the seeds look charred, most were complete and given that modern seeds are also black, there is a chance that they are modern. Two tentative identifications of fragments of the aquatic foxnut (*Eurayle* *ferox* Salisb.) were also made.

**Site 12D16**

Small charred fragments of walnut and apricot stone were the most common type of charred remains, and present within 20 and 19 of the samples respectively. Walnut fragments were most frequent in samples from Areas I and C with relatively few remains recorded within Area H (Tables S4 and S5). Fragments of walnut were classified through three main criteria; firstly, the fragments were generally thicker than other nut fragments; secondly, they possessed a vitrified, or glassy appearance, seen in the broken cross sections; and thirdly, in several cases the outer surface had a sharply undulated or a deep, but smooth pitted surface characteristic of *Juglans mandshurica* Maxim. In many instances these fragments were extremely small and absolute identification was difficult. Fragments of apricot stone were more frequent within Area H and Site 12D56. Remains of walnut from Area I (Unit I-943; sample 9139) were sent for radiocarbon dating, along with fragments of apricot stone from 12D16 Area H 805D (8142).

| **Species** | **科/属/类** | **Ubiquity** | **Ubiquity %** | **No. of seeds** |
| --- | --- | --- | --- | --- |
| *Juglans mandshurica* (frgs) | 胡桃楸 hu tao qiu | 3 | 21.43 | 1 |
| *Chenopodium* charred | 藜属 | 2 | 14.29 | 4 |
| *Nelumbo nucifera* | 莲 lian | 2 | 14.29 | 80 |
| Pyrus/Malus/Sorbus embryo/radicle | 秋子梨 | 1 | 7.14 | 0 |
| *Armeniaca vulgaris* / *sibirica*/ *mandshurica* stone | 杏/山杏/东北杏 | 5 | 35.71 | 9 |
| Trifolieae type indet. (Fabaceae) | 豆科 (未鉴定的种) | 1 | 7.14 | 1 |
| *Phellodendron amurense* (seeds) | 黄檗 (种子) | 3 | 21.43 | 1 |
| *Phellodendron amurense* (fruit) | 黄檗 (果和) | 1 | 7.14 | 0 |
| *Ampelopsis* cf. *glandulosa* | 蛇葡萄 | 2 | 14.29 | 1 |
| Paniceae indet. | 黍族 | 4 | 28.57 | 5 |
| *Panicum ruderale*/*miliaceum* | 野生稷/稷 ((黍)) | 6 | 42.86 | 5 |
| *Echinchloa* sp. | 稗属 | 1 | 7.14 | 1 |
| *Setaria italica* | 粱 (粟) | 3 | 21.43 | 2 |
| *Setaria viridis* | 狗尾草 | 2 | 14.29 | 1 |
| *Schoenoplectus* type/*Eleocharis* etc | 水葱属 | 1 | 7.14 | 1 |
| Seeds indet. | 种子 (未鉴定的种) | 2 | 14.29 | 0 |
| Nut fragments | 简而言之 | 2 | 14.29 | 4 |
| Fruit/Nut fragments indet. | 核 /简而言之 (未鉴定的种) | 3 | 21.43 | 3 |
| Large frg. fruit / tuber |  | 2 | 14.29 | 1 |

**S4 Table: Flotation results of Site 12D16 area H**

| **Species** | **科/属/类** | **Ubiquity** | **Ubiquity %** | **No. of seeds** |
| --- | --- | --- | --- | --- |
| *Phellodendron amurens*e (seeds) | 黄檗 (种子) | 3 | 5.56 | 1 |
| *Phellodendron amurens* (fruit) | 黄檗 (果和) | 3 | 5.56 | 3 |
| *Onagraceae/Boraginaceae*. Small seeds  indet. |  | 3 | 5.56 | 1 |
| *Hyocyamus/Lycium/Physaliastrum* | 天仙子/枸杞/日本散血丹 | 4 | 7.41 | 0 |
| Culm node (Poaceae) |  | 3 | 5.56 | 1 |
| Paniceae indet. | 黍族 | 4 | 7.41 | 0 |
| *Panicum ruderale/miliaceum* | 野生稷/稷 ((黍)) | 5 | 9.26 | 3 |
| Cyperaceae small tuber | 莎草科 | 3 | 5.56 | 1 |
| *Schoenoplectus* type/*Eleocharis* etc | 水葱属 | 3 | 5.56 | 3 |
| Small seed indet. | 小种子 (未鉴定的种) | 3 | 5.56 | 1 |
| Small indet.termite pellets |  | 3 | 5.56 | 25 |
| Seeds indet. | 种子 (未鉴定的种) | 5 | 9.26 | 5 |
| Nut fragments | 简而言之 | 4 | 7.41 | 0 |
| Fruit/Nut fragments indet. | 核 /简而言之 (未鉴定的种) | 10 | 18.52 | 46 |
| Fruit stone frags |  | 3 | 5.56 | 0 |
| Tuber/root |  | 2 | 3.70 | 0 |
| Parenchyma |  | 7 | 12.96 | 0 |
| Buds | 树花苞 | 8 | 14.81 | 16 |

**S5 Table: Flotation results of Site 12D16 area I**

A further edible nut identified within the samples was lotus seed (*Nelumbo nucifera* Gaertn.). Specimens were potentially present in several samples, however, they were only positively identified from numerous fragments within one single sample from 12D16 Area H 805D (8142). While a few specimens were present in 12D16 Area C, Site 12D56 and Area I identification of this material was less certain.

Fragments of lotus seed were considerably thinner than either those of apricot or walnut, but more specifically, while the outer surface was relatively smooth the inner surfaces of the archaeological specimens possessed a rugose pattern that compared well to modern examples. Such a pattern is similar to that seen on the outer surface of foxnut (*Euryale ferox* Salisb.), which has larger, more widely spaced tubercles. Using this distinction, a few possible fragments of foxnut (*Euryale ferox*) were also recorded within one sample from Area C and two from site 12D56, but these are only tentative identifications. Fragments of probable lotus seed from Area H (Unit 805, sample 8142) were sent for radiocarbon dating.

Fragments of fruit and seeds of Amur cork were recovered from all three excavation areas, although remains of this species were both much less well preserved and less well represented than within site 12D56. In site 12D16 Amur cork was more frequent within the samples from area H, being present in only one sample from Area I (Unit I943), with just a few fragments from Area C. The seeds of Amur cork are highly distinctive, around 5-6mm long and 2.5-3mm wide, with a characteristic, relatively large (0.2-0.3 mm diameter), hexagonal to sub-hexagonal cell pattern overlying a finer cell pattern. This characteristic allowed for identification of many smaller fragments.

Two specimens of probable porcelain-berry/snake grape or *she-putao* (蛇葡萄, *Ampelopsis* *glandulosa* (Wall.) Momiy./ 三裂蛇葡萄 *A. delavayana* Planch. ex Franch.) were found from Area H, one of which was complete (sample 8134) and one probable fragment (sample 8146). The species, which is edible, is closely related to grape (*Vitis* sp.) and the seeds also strongly resemble those of grape. However, mountain grape/*shan-pu-tao*, (山葡萄, *Vitis amurensis* Rupr.) is the only species of *Vitis* found wild in Liaoning [51]. Differences used to distinguish the charred seed from *Vitis amurensis* Rupr. were based upon the lack of a visible “notch” at the base of the seed, a shorter less prominent beak, the shape of the fossettes (the two apertures on the ventral surface) and the sharper less rounded angle of the ventral surface. Additionally, the chalaza on the dorsal surface was less visible and appeared longer and less circular than that of *Vitis amurensis*. It might be noted that three other species of *Ampelopsis* also occur in Liaoning, *A. aconitifolia* Bunge, *A. humulifolia* Bunge and *A. japonica* (Thunb.) Makino [51] but from the observed morphology of these seeds all seem less likely candidates.

Also recovered from Area C (Unit C304, 3008) was a single stone of mountain hawthorn, (*shan zha* 山楂) *Crataegus* cf. *pinnatifida* Bunge. As with many of the other species listed above, the fruit is edible, and today there are cultivated varieties known as *shanlihong* (山里红) or “Mountain Red”. This stone was sent for radiocarbon dating.

A radical/embryo tip of a Rosaceae (tribe Maloideae) seed, probably of apple (*Malus bacatta* (L.) Borkh., *Malus mandshurica* (Maxim.) Kom. ex Juz. Or *Malus prunifolia* (Willd.) Borkh.), pear (*Pyrus ussuriensis* Maxim. Ex Rupr. or *Pyrus betulifolia* Bunge) or *Sorbus* (*Sorbus esserteauiana* Koehne, *Sorbus pohuashanensis* (Hance) Rehder or *Sorbus alnifolia* (Siebold & Zucc.) K.Koch) was recorded from Site 12D16 Area H (sample 8146). The small size, even taking into account charring, would seem more in keeping with *Sorbus* than apple and pear, however the embryo/radical scar is often smooth on seeds of species of *Sorbus*, unlike the charred specimen. It might be noted that all of these species are edible.

**Millet**

Relatively few millets were recorded within the samples. However, of those recovered, the majority, 16 grains, were of common millet (*Panicum miliaceum* L.), and in most cases these were small and more similar in size to wild-type Panicum (*Panicum miliaceum* subsp. *ruderale* (Kitag.) Tzvelev) than modern domesticated broomcorn e.g. on average 1.25-1.70mm in length and 1.05-1.40 in width. Generally, only single grains were recorded from individual samples; two from Area C (C303), three from Area I; along with one immature and nine potential grains from Area H.

Grains of foxtail millet (*Setaria italic* (L.) P.Beauv.) were less common and only a single positively identified grain was recorded within a sample from Area H, along with two grains of green foxtail (*Setaria viridis* (L.) P.Beauv.), and two grains of wild or cultivated *Setaria* sp. from three further samples. A single grain of *Echinochloa* sp. was also recovered from Area H. A number of unidentified Paniceae were also recovered from the samples, some potentially of *Panicum* *miliaceum/ruderale*.

The samples also included stems and occasional culm nodes of Poaceae. Also present, indicative of wetlands, were small possible tubers of sedges (Cyperaceae) from Area H, a seed of probable *Scirpus / Schoenoplectus* from Area I, and an inner seed kernel of a Cyperaceae nutlet from Area H (sample 8142).

Other recorded seeds included those of *Chenopodium* and *Atriplex*, both in the Chenopodiaceae. *Atriplex* was represented by a single charred seed, while several seeds of *Chenopodium* sp. were also recorded, mainly from Area C. While some of these seeds were easily ascertained as being charred, in a few cases recorded in S1-S4 Tables it was not possible to ascertain that they were not uncharred. Clear examples of uncharred modern seeds of *Chenopodium* were relatively common in several of the samples, especially those with high numbers of roots. Several seeds (*c.* 1.30-1.5 mm x 1.10-130mm) of an unidentified Solanaceae came from Pit C. The cell pattern is relatively large, in comparison to many genera within this family, especially *Solanum*. The *Flora of China* [51] lists 10 native genera, however only three are comparable in size and cell patterns to those found in northeast China. Goji /wolfberry (枸杞, *Lycium chinense* Mill.) is cultivated today for its berries, but also grows as a general weed of waste places. Like goji, *tian xian zi* (天仙子; henbane, *Hyoscyamus niger* L.), is also cultivated but predominately for medicinal purposes, but otherwise is a common weed species. *Ri ben san xue dan* (日本散血丹, *Physaliastrum echinatum* (Yatabe) Makino) is only known as a weed more common on grassy slopes and woodland edge [48].

A further seed of a small Fabaceae was recovered from Area I. The seed in several aspects resembles wild soybean (*Glycine soja* Siebold & Zucc.) although unfortunately the hilum is absent. However, its small size, 1.4mm long and 0.8mm wide, would put it with the smallest charred examples recorded archaeologically [see 18,53]. A further seed, that resembles a large Chenopodiaceae, was distinctive in having a very thick seed coat, and in this respect was uncharacteristic of the Chenopodiaceae, and more similar to seeds of species such as *Menyanthes trifoliata* L. or more probably *Nymphoides indica* (L.) Kuntze/ *coreana* (H. Lev.) H. Hara, all associated with standing water bodies.

A number of further unidentified seeds were also recovered, although in most cases these comprised single instances. Two further possible specimens of a similar Boraginaceae- type seed to that recovered from 12D56 were also present in Area I, 9062 and 9072. The seeds are just under 1mm long and around 0.68mm wide, with a distinctive surface texture, closely resembling in shape, but not size, the seeds of *Lithospermum*, *Echium* etc. but are similar also to species within the Rutaceae or within the Onagraceae.

Several very small sub-oval seed type objects (*c*. 0.5mm in length) were also recovered from Area I (sample 9062). These compare well to termite frass pellets, but also to small seeds, such as *Mitella nuda* L. or *Ludwigia epilobioides* Maxim. The latter are, however, more characteristic of wet places, such as ditches and stream sides. Modern seeds also often have a distinct shallow ridge that runs down the length of the seed, which should be visible on at least some of the ancient specimens, but appears absent. As such it is suspected presently that these objects are more likely termite pellets than seeds.

Samples from Site 12D16 (Area I) were by far the richest in wood charcoal, as was expected from samples recovered near burnt wooden beams. Samples form Site 12D16 Area C also had wood charcoal but the other samples had relatively little to no wood charcoal within them. Wood charcoal often fractures along the longitudinal planes and so is particularly affected by bioturbation. This was most evident in Area I, where samples from contexts that were seen in the field to be relatively charcoal rich, still produced little to no charcoal after flotation. This was especially the case in samples where large numbers of roots were still present, comprising 80-90% of the flot volume, despite the sampled deposits lying below the modern visible root zone. The material from Area I included charcoal that was ring-porous (e.g. samples 9009 and 9053) and particularly noted is sample 9143, which contained several fragments of round wood of around 1cm diameter.

Finally, in addition to wood charcoal, there were also several examples of tree buds. These came mainly from area I, but also from area H, and may be of leaf or flower buds. As with the finds of small round wood, these would be in keeping with the burning of smaller branch material.

**Bioturbation and modern contamination at the sites**

Charred material was relatively sparse in the flotation samples, and some of the samples yielded a large number of roots and modern seeds, indicative of bioturbation and hence possible contamination. This was most notable in the uppermost samples from Site 12D16 (Area H) and Site 12D56. In one sample from Site 12D16 Area H (8044), partially charred material was seen, including a burnt grass stem, which raises the possibility of modern contamination. A number of samples had in them modern plant material, including seeds of *Portulaca oleracea* L., *Amaranthus* sp., *Chenopodium* sp. and *Acalypha australis* L., all of which are externally black and therefore not easy to distinguish from ancient charred material until broken. Other modern material included large numbers of uncharred grass seeds of green foxtail (*Setaria viridis*) and *Digitaria* sp., along with seeds of *Potentilla* sp., *Mosla* sp., *Mentha/Clinopodium* sp., *Ajuga* sp. and *Viola* sp.. Uncharred material from modern cultivation included spikelets of *Setaria italica*, peanut (*Arachis hypogaea* L.) shells, and seeds of maize (*Zea mays* L.), although all of these were relatively uncommon.

**Radiocarbon Dating**

The results of the radiocarbon dating on plant macrofossils are shown in S3 Table. In general the results are in good agreement with those on wood charcoal. The results from Site 12D16, Area C are highly similar to those from Area I, and in broad terms in agreement with the wood charcoal dates, indicating a period of occupation between 5620-5480 cal. BCE. The date on apricot stones from site 12D16 Area H is broadly contemporary with that on hawthorn from Site C and walnut from Area I, although statistically the dates are not contemporary, being slightly younger. This again is consistent with the dates on wood charcoal from area H, which are of a similar date, but of slightly younger date than the wood charcoal dates from Area I. As also seen in the results obtained on wood charcoal, those from Area 12D56 appear a few centuries older, providing a date between 5990-5790 cal. BCE, comparable with the older wood charcoal dates.

The date on possible *Nelumbo* from site 12D16 Area H, is not consistent with the other dates, and at face value appears contemporary with the Eastern Zhou period (790-540 BCE), and therefore probably contains or comprises entirely of intrusive material. The samples came from the upper fills of features within this area that were heavily bioturbated. In addition, *Nelumbo nucifera* was only present within Area H. However, given five fragments of *Nelumbo* were submitted, there is a possibility that the material may present a mixture that includes Xinglongwa and modern material, hence yielding an average date of 790-540 BCE, rather than representing activity directly relating to the Eastern Zhou period from which no identified artifacts (potshards) are known in this site. Unfortunately, this date does raise the possibility that some other material within this area, including smaller millet seeds and mountain grape, may be intrusive from later activity upon the site.

**Acknowledgements**

A large portion of the samples were floated by Ms. Wang Huan. This paper was supported by a European Research Council grant “Comparative Pathways to Agriculture” (ComPAg, no. 323842). Work in Beijing at Peking University was facilitated through ICCHA (中国文化遗产保护与考古学研究国际中心; International Centre for Chinese Heritage and Archaeology).

**S1f. δD_wax_ data from the two archaeological sites**

To evaluate changes in the isotopic composition of precipitation, we analyzed the hydrogen isotope composition of long-chain *n*-alkanes extracted from sediments in both archaeological sites. Long-chain *n*-alkanes are produced in leaf-waxes of terrestrial plants and their hydrogen isotopic composition (δD_wax_) is primarily governed by the isotopic composition of precipitation [38].

Site 12D56 is composed of a single structure, dug into the natural soil to a depth of >20 cm. We sampled a section that cuts through the structure (S3 Fig). A1 is the natural sediment below the archaeological horizon, which must be older than the occupation (i.e. older than ~7900 Cal BP). A2 is the archaeological sediment. A3 is the topsoil, which is younger than the occupation horizon and probably represents modern conditions.

**S3 Fig. δD_wax_ sampling location in Area A of site 12D56**. The structure was dug into natural soil that sits on top of granite bedrock. The samples (A1-A3) were sampled in the natural soil, archeological material and the modern topsoil.

In site 12D16, we sampled two structures from areas I and H. The structure in Area H was dug into the natural bedrock to a depth of >60 cm. Archaeological sediments fill the structure and thus provide a 60 cm section (S4 Fig). We sampled this section in detail, following the stratigraphy and archaeological horizons found during the excavation. H1 was sampled in the natural sediment below the boundary of the structure, which must be older than the occupation (i.e. older than ~7500 Cal BP). H3 and H5 were sampled from within the archaeological material. H8 was sampled from the topsoil, which is younger than the occupation horizon and probably represents modern conditions.

**S4 Fig. δD_wax_ sampling location in Area H**. The top figure shows a profile of a structure dug into the natural soil, which is filled with archaeological material. The bottom figure shows a close-up of the sampling locations of δD_wax_ (H1, H3, H5 and H8) and results of radiocarbon samples as a function of depth.

Area I is composed of a single rectangular structure, which was burnt and contains many archaeological remains on the floor of the structure. We sampled sediments from the ash layer (I-3) (S5 Fig).

**S5 Fig. δD_wax_ sampling location in Area I**. The sample (I-3) was sampled in the burnt layer associated with the broken vessels lying on the floor of the structure.

*Laboratory procedures.* The sediments were lyophilized overnight (-55˙C at 10 millitorr) and all macro-organics were picked out. The total lipid extract (TLE) was extracted from ~30 g of sediment using a Dionex Accelerated Solvent Extractor (ASE 350). Samples were extracted with 9:1 dichloromethane:methanol (DCM:MeOH, v/v) for the extraction and four 10-min static cycles at 100˙C with a flush volume of 150%.

To trace possible sample loss throughout the procedure and to quantify compound concentration, 2000 ng each of laboratory recovery standard (5-α-androstane) was added the samples. The extraction solvent was evaporated under a stream of N_2_ and the TLE separated into hydrocarbon, ketone and polar fractions using silica gel columns. The column was prepared in a Pasteur pipette with a DCM-rinsed glass wool plug and 0.5 g of silica gel (60 Å pore size, 70-230 mesh). Hydrocarbons were eluted from the column with 4 mL of hexane; ketone, ester, and aromatic compounds were eluted with 4 mL of dichloromethane; and polar compounds were eluted with 4 mL of methanol. The hydrocarbon fraction containing the *n*-alkanes and the polar fraction containing the fatty acids were used for further analysis.

*n*-alkanes were analyzed in the Lamont-Doherty Earth Observatory Organic Geochemistry Laboratory. Relative abundances of *n*-alkanes were determined on an Agilent 7890A GC equipped with both a mass selective detector (5975C MSD) and flame ionization detector (FID). A multi-mode inlet (MMI) operated in pulsed temperature vaporization (PTV) mode, and a 30m, HP-5 column were used to separate compounds. One microliter of the sample in hexane was injected into the MMI at 60˙C. The inlet was ramped at 999C/min to 320˙C. The oven was held at 60˙C for 1 minute then ramped at 22˙C /min to 200˙C and then 4 ˙C /min to 320 followed by a 10 minute hold. Compound abundances were quantified using the MSD peak area normalized to the 5-α-androstane internal standard. We corrected for mass-dependent changes in ionization efficiency using a standard mixture of C8-C40 *n*-alkanes.

*Hydrogen isotope measurements.* Hydrogen measurements were conducted at the Organic Geochemistry Laboratory at Lamont Doherty Earth Observatory on a Thermo DeltaV coupled to a Thermo Trace GC through a Thermo IsoLink and ConFlo IV. Hydrogen isotope analysis was conducted using a pyrolysis reactor (empty alumina tube) at 1420˙C conditioned with two 1 μl injections of hexane [54]. Evolved H_2_ gases are introduced to the IRMS through a continuous flow device. A calibrated laboratory reference gas was injected before and after each run, and used to assess isotope drifts throughout the run. A molecular mixture with known isotopic values (mix A5 supplied by Arndt Schimmelmann, Univ. of Indiana) was injected between groups of 5-10 samples and used to correct the isotope values to the VSMOW scale. Thermo ISODAT software was used to calculate the isotope value with respect to the reference gas injections. Isotope ratios and analytical uncertainty were calculated using [55]. In short, the method calculates the measurements for isotope drifts throughout the life of the reactor, converts the values to VSMOW scale and calculates the total uncertainty of the measurements.

To assess hydrogen isotope drift throughout the life of the reactor, a ‘drift’ sample was run a few times at the beginning of a reactor and then between every 5 samples. The isotopic values of a new reactor stabilize after ≈ 5-10 injections. Throughout the run, the isotopic value of nC29 and nC31 in the ‘drift’ sample was monitored and compared to the stabilized isotopic value. Samples were corrected based on the drift trend of nC29 and nC31 in the ‘drift’ sample from the stabilized isotopic value. H^+^_3_ factor was run between every 5-10 samples and samples were corrected accordingly. Hydrogen samples were converted to VSMOW scale using the average isotope values of all compounds in the Mix-A5 standard (supplied by Arndt Schimmelmann, Indiana University, Bloomington, IN). The standard deviation of the mean of the reference gas molecules was larger than the analytical [Case 2 in ref 55], therefore, laboratory reference gas uncertainty is quantified using the standard deviation of the means of the reference gas uncertainty. For hydrogen, we used GC peak areas ranging 20-300 V/s. These values were chosen because they yielded the lowest uncertainty in a series of peak size evaluations (±4.5 ‰ for C29). To assess the δD_wax_ from the archaeological sites we calculated the unweighted average of C25-C33 (^avg^δD_wax_). Due to analytical problems (large peak size), sample H_3_ was excluded.

The results show that the earlier site (12D56) has an ^avg^δD_wax_ (-165 ‰), which is more enriched than the later sites (12D16) (-186‰). Based on the empirical correlation between the isotopic composition of precipitation and local rainfall in north China during the Early and Middle Holocene [32], the isotopic composition can be used as a qualitative proxy for rainfall amount. The more depleted values in the later site relative to the early sites, indicates that rainfall amount was higher during the later site relative to the early site (S6 Fig and Table S6). Due to the large uncertainty in the conversion of δD_wax_ to rainfall δD [55] and the uncertainty in the relation between rainfall δD and rainfall amount, these results should be viewed as qualitative.

**S6 Fig. δD_wax_ results from the two sites**. The earlier site (12D56) is in purple; the two structures from the later period are in orange (area H) and blue (area I). The samples that predate the site were assigned an arbitrary age of 9500 BP to prevent confusion, but the only age constraint we have is that they are older than the archaeological occupation.

**
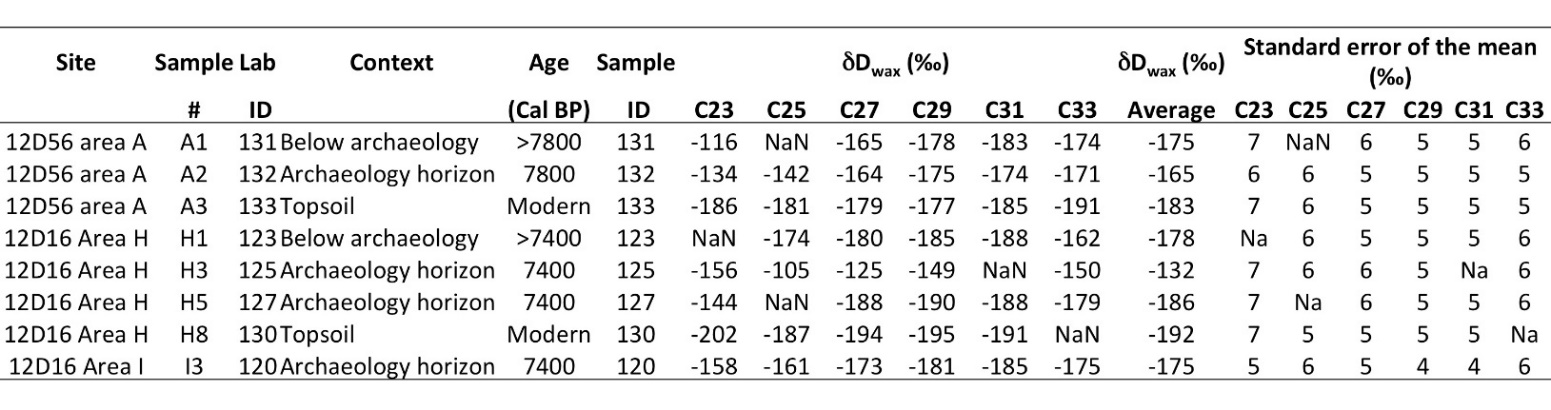
**

**S6 Tab. δD_wax_ results from the two sites**

**Additional References**

42. Ramsey CB, Lee S. Recent and Planned Developments of the Program OxCal. Radiocarbon. Cambridge University Press; 2013;55: 720–730. doi:10.1017/S0033822200057878

43. Reimer P, Bard E, Bayliss A, Beck J, Blackwell P, Ramsey C, et al. Intcal13 and marine13 radiocarbon age calibration curves 0 – 50,000 years cal bp. Radiocarbon. 2013;55: 1869–1887. doi:10.2458/rc.v51i4.3569

44. Maeda O, Lucas L, Silva F, Tanno KI, Fuller DQ. Narrowing the harvest: Increasing sickle investment and the rise of domesticated cereal agriculture in the Fertile Crescent. Quat Sci Rev. 2016;145: 226–237. doi:10.1016/j.quascirev.2016.05.032

45. Fuller DQ, Qin L. Declining oaks, increasing artistry, and cultivating rice: the environmental and social context of the emergence of farming in the Lower Yangtze Region. Environ Archaeol. Routledge; 2010;15: 139–159. doi:10.1179/146141010X12640787648531

46. Ishikawa S. Seeds and Fruits of Japan (原色日本植物種子写真図鑑). Japan: Ishikawa Shigeo Publication; 1994.

47. Institute of Botany Chinese Academy of Sciences (IBCAS). On-line Seed Database [Internet]. 2017.

48. Liu C, Jin G, Kong Z. Archaeobotany: Research on seeds and fruits (植物考古-种子果实研究 ). Shandong: University Orient Archaeology Research Series. Beijing: Science Press (in Chinese); 2008.

49. Nakayam S, Inokuchi M, Minamitani T. Seeds of Wild Plants in Japan (日本植物種子图鉴). Sendai (in Japanese): Tohuku University Press:; 2000.

50. Suzuki T, Takahashi F. Seeds and Fruits Illustrated (草木の種子と果実―形態や大きさが一目でわかる植物の種子と果実632種). 2012.

51. Wu Z, Raven PH, Hong D. Flora of China. St. Louis: Science Press, Beijing, and Missouri Botanical Garden Press; 2001.

52. (アイヌ民族博物館) AFM. Entry for Phellodendron amurense / Kihada, Karafuto Kihada [キハダ カラフトキハダ] / Shikerepeni [シケﾚペニ] in Ainu Digital Nature Books [アイヌと自然デジタル図鑑]. In: http://www.ainu-museum.or.jp/siror/book/detail.php?page=book&book_id=P0172. Ainu Digital Nature Books; 2017.

53. Lee G-A, Crawford GW, Liu L, Sasaki Y, Chen X. Archaeological Soybean (Glycine max) in East Asia: Does Size Matter? Newsom LA, editor. PLoS One. Public Library of Science; 2011;6: e26720. doi:10.1371/journal.pone.0026720

54. Burgoyne TW, Hayes JM. Quantitative Production of H 2 by Pyrolysis of Gas Chromatographic Effluents. Anal Chem. 1998;70: 5136–5141. doi:10.1021/ac980248v

55. Polissar PJ, D’Andrea W. Uncertainty in paleohydrologic reconstructions from molecular δD values. Geochim Cosmochim Acta. Elsevier Ltd; 2014;129: 146–156. doi:10.1016/j.gca.2013.12.021
